# Surge arrester leakage current modeling based on pollution layer electrical conductivity estimation

**DOI:** 10.1038/s41598-025-29594-y

**Published:** 2025-11-27

**Authors:** Arian Hoseini Nejadiyan Kooshki, Seyydmeysam Seyyedbarzegar

**Affiliations:** Faculty of Electrical Engineering, Shahrood University of Technology, Shahrood, Iran

**Keywords:** Metal oxide surge arresters, Pollution conductivity, Leakage current, Uniform and non-uniform pollution, Random Forest, Electrical and electronic engineering, Characterization and analytical techniques, Software

## Abstract

The surface leakage current (LC) of metal oxide surge arresters (MOSA) is highly dependent on environmental conditions. This factor influences the modeling of MOSA leakage current as an alternative solution to laboratory tests. In this paper, a new approach to modeling the surface leakage current of MOSA based on the estimation of the electrical conductivity (EC) of the contaminated layer is presented, which provides the ability to model the surge arrester LC in different environmental conditions with high accuracy. The estimation of the EC of the polluted layer has been performed using artificial intelligence (AI) based on laboratory tests considering the effect of uniform and non-uniform pollution, humidity, pollution intensity and voltage on three types of 20 kV silicon rubber surge arresters. Mean Squared Error (MSE) and Coefficient of Determination were used for assessing the ability of AI based method in EC estimation. Finite element method (FEM)-based software has been used for surge arrester modeling. The use of the estimated electrical conductivity characteristic in the FEM model has made it possible to evaluate the effect of the internal and external currents of the MOSA on the total leakage current in different scenarios. The comparison of the results obtained from proposed model and laboratory test indicate the capability of the proposed method in estimating the EC, modeling the LC, and their generalization to cases for which laboratory test results are not available.

## Introduction

Metal oxide surge arresters are one of the most widely used devices that protect the network against switching and lightning overvoltage. Considering the very important role of MOSAs in the power network, it is very important to know their status at any moment. Monitoring and supervision of the MOSA is done in several ways, some of these methods are destructive and some are non-destructive. Among them the power loss method^1,2^, voltage-current (V-I) characteristic curve analysis method^3^, temperature measurement and control method^4,5^, electromagnetic field measurement method^6,7^, Remote surge arrester monitoring device^8^ and leakage current measurement^9–12^ can be mentioned. The leakage current of the MOSA contains very important information, whose extraction and analysis have always been of interest to researchers and industry. In fact, the method of measuring the leakage current is one of the suitable and non-destructive methods for monitoring the MOSA.

Under normal conditions, the MOSA does not show any operation and is like a very large impedance. Under these conditions, the leakage current passes through the MOSA, which is obtained from the combination of the current passing through the varistors and the body of the MOSA^12^. The varistor voltage-current characteristic and environmental conditions as an effective factor on MOSA performance have a great impact on leakage current. In fact, the V-I characteristic of the MOSA’s varistors may change during the operation period. Also, due to the installation of MOSAs in outdoor, environmental conditions such as pollution and humidity (H) will affect the leakage current and change its amplitude and harmonic components^13^.

The use of leakage current in MOSA monitoring requires the separation of its resistive and capacitive components from the total leakage current. The resistive component of the leakage current has changed under the influence of environmental conditions, which can provide very good information about the conditions of the MOSA^14^. Research has shown that pollution may be placed on the insulation surface of the equipment in a uniformly and non-uniformly manner^15^. Non-uniform longitudinal and fan-shaped type contamination may appear on the MOSA. The occurrence of non-uniform contamination as a factor dependent on environmental conditions strongly affects the leakage current parameters of the MOSA^16^.

In order to measure the leakage current and analyze it, it is necessary to examine the MOSA in a laboratory and measure the leakage current signals under different environmental and operating conditions. Providing laboratory conditions requires a lot of effort and time, which must be done with great care. Also, as a result of leakage current passing over the insulating surface, a dry band may be created, which leads to a surface defect. Therefore, the MOSA modeling approach can be considered as an alternative solution. The use of software based on finite element method has the ability to simulate the environmental conditions with high precision in addition to the accurate modeling of the MOSA. In general, by developing an accurate model capable of simulating laboratory results, it is possible to obtain the leakage current under different environmental conditions that have not even been measured in the laboratory environment^14^.

Although the modeling approach is a suitable solution to save money and time compared to laboratory testing, the results are dependent on the initial conditions. Choosing a MOSA with specific dimensional parameters, V-I characteristic of varistor, pollution layer and its electrical conductivity are among the initial conditions for each mode of simulation. Changing any of these parameters will result in a different output. Meanwhile, the electrical conductivity of the contaminated layer has the greatest impact on the amplitude and harmonic components of the modeled leakage current due to the non-linearity of its voltage-current characteristic. Therefore, the use of a suitable method with acceptable accuracy to reduce the sensitivity of modeling to this parameter should be considered. The estimation approach as a reliable tool has the ability to produce the electrical conductivity of the contaminated layer in a wide range of initial conditions. Due to the simultaneous effect of several factors on the leakage current and the extent of laboratory conditions, the use of methods based on artificial intelligence helps a lot in order to achieve the stated goal. In^17^, an improved electrical model for metal oxide surge arrester considering the effects of temperature is presented. In this model, an artificial neural network is used to estimate the V-I characteristic. Also, thermal equilibrium diagram of MOSA has been studied in^18^ using an adaptive network fuzzy inference system. For insulator modeling, the ANN is used in^19^ to estimate the leakage current values based on pollution layer parameters.

**Fig. 1:**
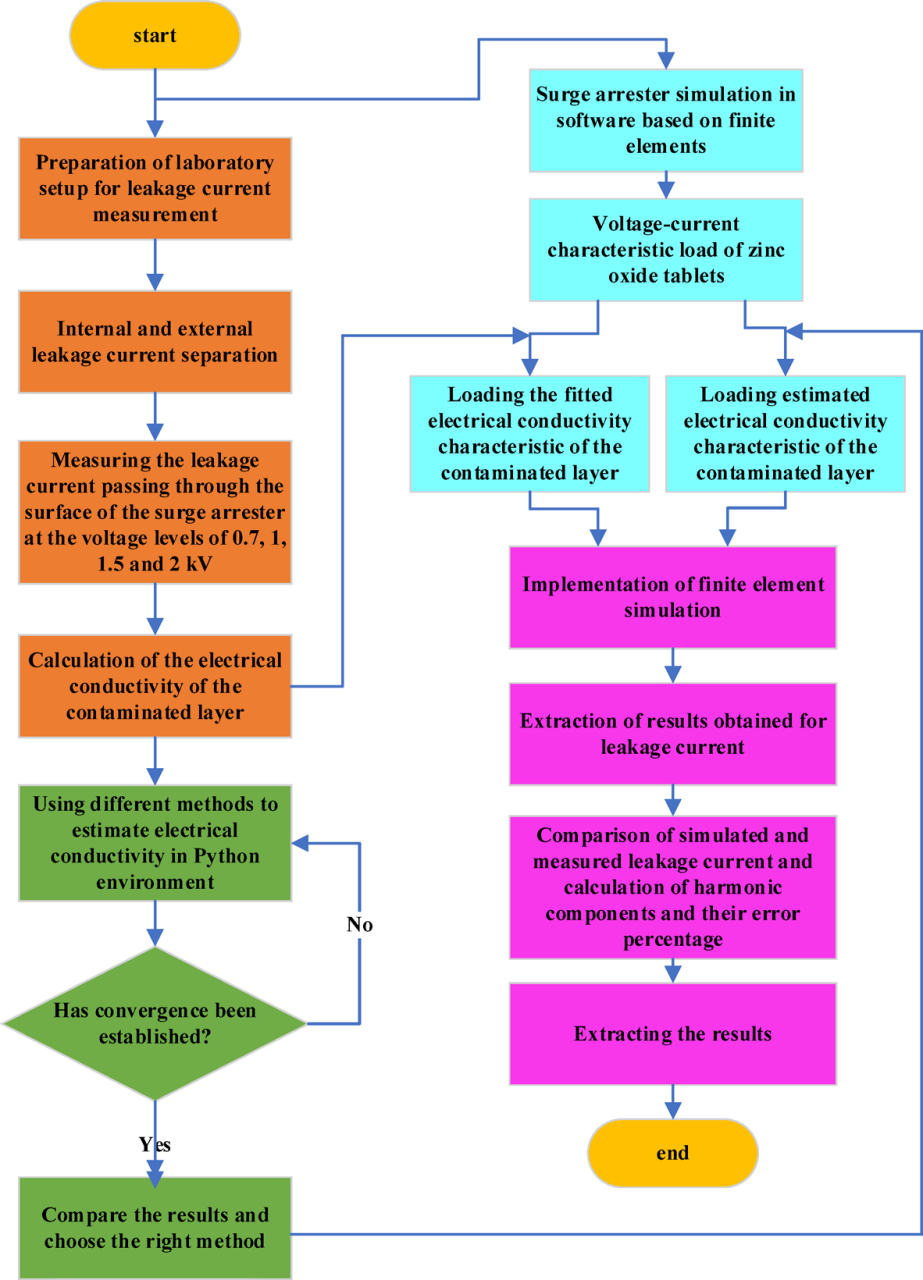
Flowchart of the process of modeling the electrical conductivity of the contaminated layer and simulating the leakage current.

In this paper, a new method for modeling the leakage current of MOSAs under different environmental conditions is proposed. Modeling was done for three types of 20 kV silicon rubber MOSAs. Considering the importance of electrical conductivity of the contaminated layer in changing the leakage current, an estimation model was created based on the artificial intelligence method using python language. Since the total leakage current of MOSA consists of the current passing through the surface and varistors, one of the goals of this research is to replace the estimation of the leakage current with the EC estimation of the contaminated layer. With this estimate, the total leakage current can be modeled in a wide range of pollution and environmental conditions changes without the influence of factors such as the structure and V-I characteristics of the MOSA. In this article, MOSA was modeled in FEM-based software so that in addition to electrical parameters, its dimensional and structural conditions can also be changed. In order to validate the results obtained from the presented model, the leakage current was measured under different environmental conditions, experimentally. The laboratory tests on the MOSAs with different dimensional parameters for two kinds of uniform and fan-shaped type non-uniform pollution were carried out. The process of estimation of the contaminated layer EC and simulation of the leakage current is shown as a flowchart in Fig. 1.

## Laboratory test

In order to measure the pollution layer conductivity, leakage current of the MOSA and investigate the effect of environmental factors such as humidity and pollution, the laboratory setup was prepared as follows.

### Laboratory setup

**Fig. 2:**
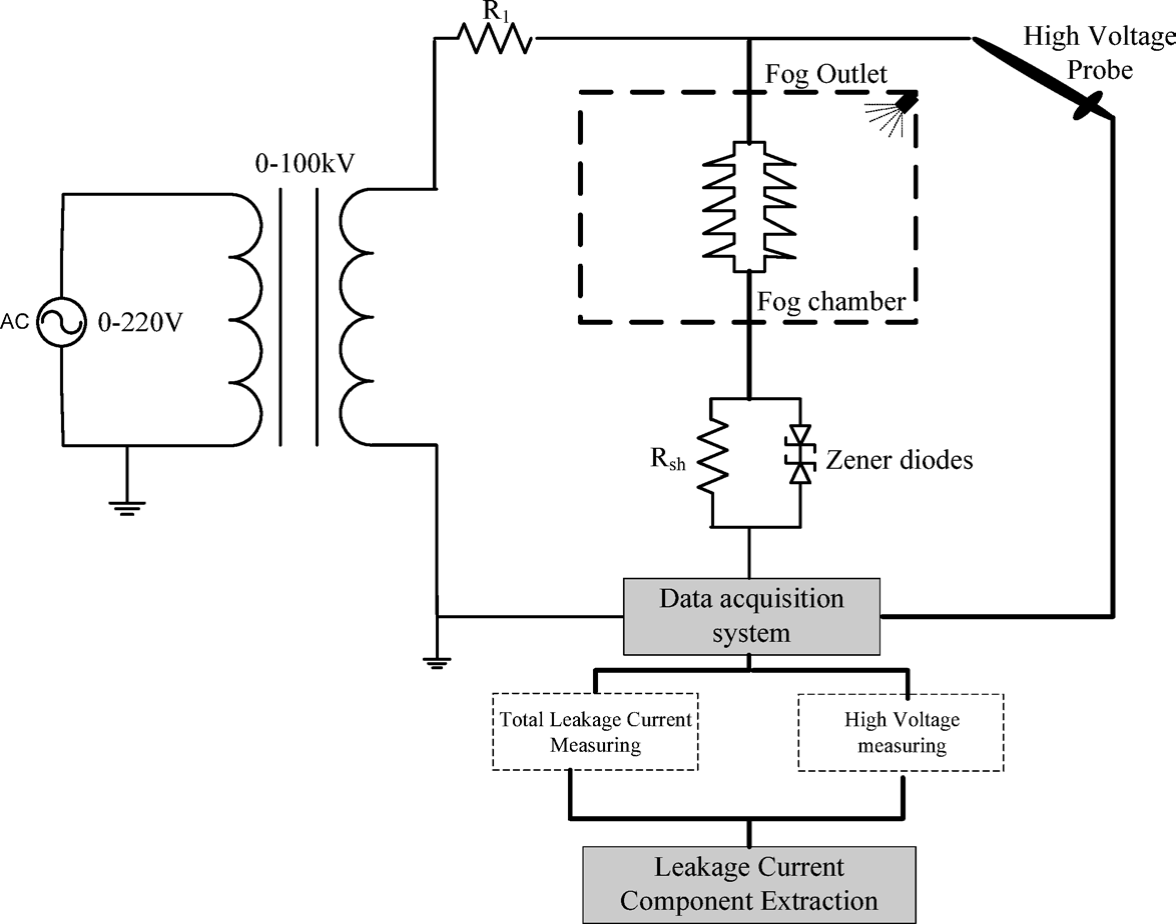
High voltage laboratory setup.

**Fig. 3:**
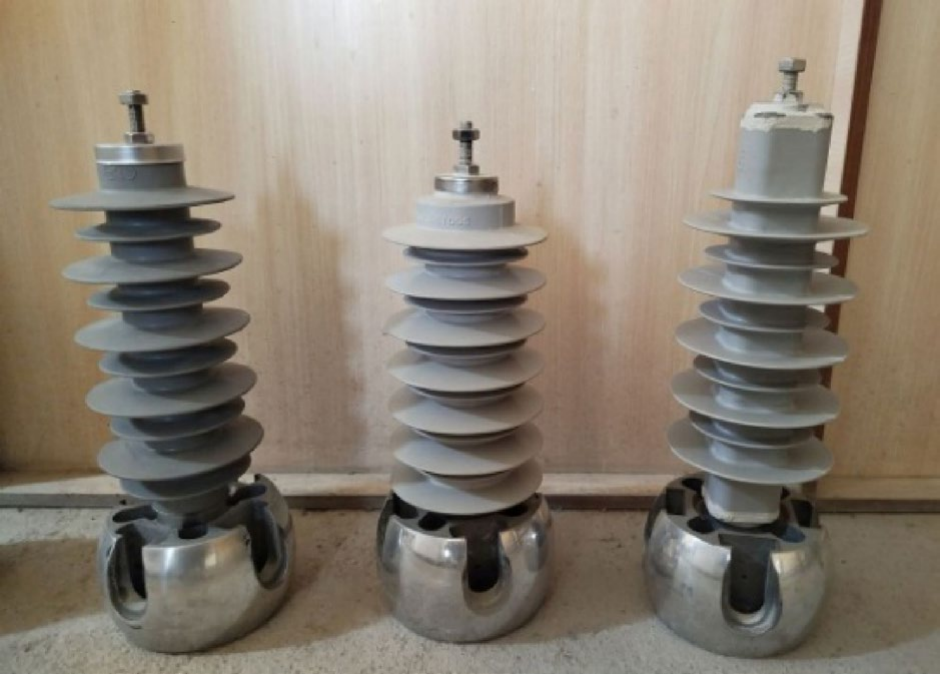
Silicon rubber MOSAs.

The high-voltage laboratory setup includes a 220 V/100 kV transformer whose output can be adjusted between 0 and 100 kV. To ensure protection of the transformer against short circuit current, a 10 MΩ protective resistor (R1) is placed in series with the source. To measure the leakage current, Rsh resistance is used in series with the MOSA, and the measured information is recorded and stored through a digital scope. The applied voltage is measured through a high voltage probe. The high-voltage laboratory setup is shown in Fig. 2. In all experiments, high-purity distilled water was used in order to minimize the effect of external factors on the electrical conductivity of the contaminated layer. To check the performance of the MOSA in different weather conditions, fog maker and fog chamber with dimensions of 2 * 2 * 2 m have been used. For measuring process, three types of silicone rubber surge arresters with different structural characteristics have been provided at the voltage level of 20 kV according to Fig. 3. The electrical characteristics of these MOSAs are given in Table 1.

**Table 1:** Electrical characteristics of MOSAs.

Type	Type 1	Type 2	Type 3
Total height of MOSA (cm)	26	28	31
Maximum diameter of shed (cm)	12.2	11.5	11.2
Shed numbers	13	10	10
Total external creepage distance (mm)	810	740	750
Rated voltage (kV)	25	25	25
Continuous operating voltage (kV)	20	20	20
Lightning impulse current 8/20 µs (kA)	10	10	10
Maximum residual voltage (kV)	75	70	70

### Uniform and non-uniform pollution

**Fig. 4:**
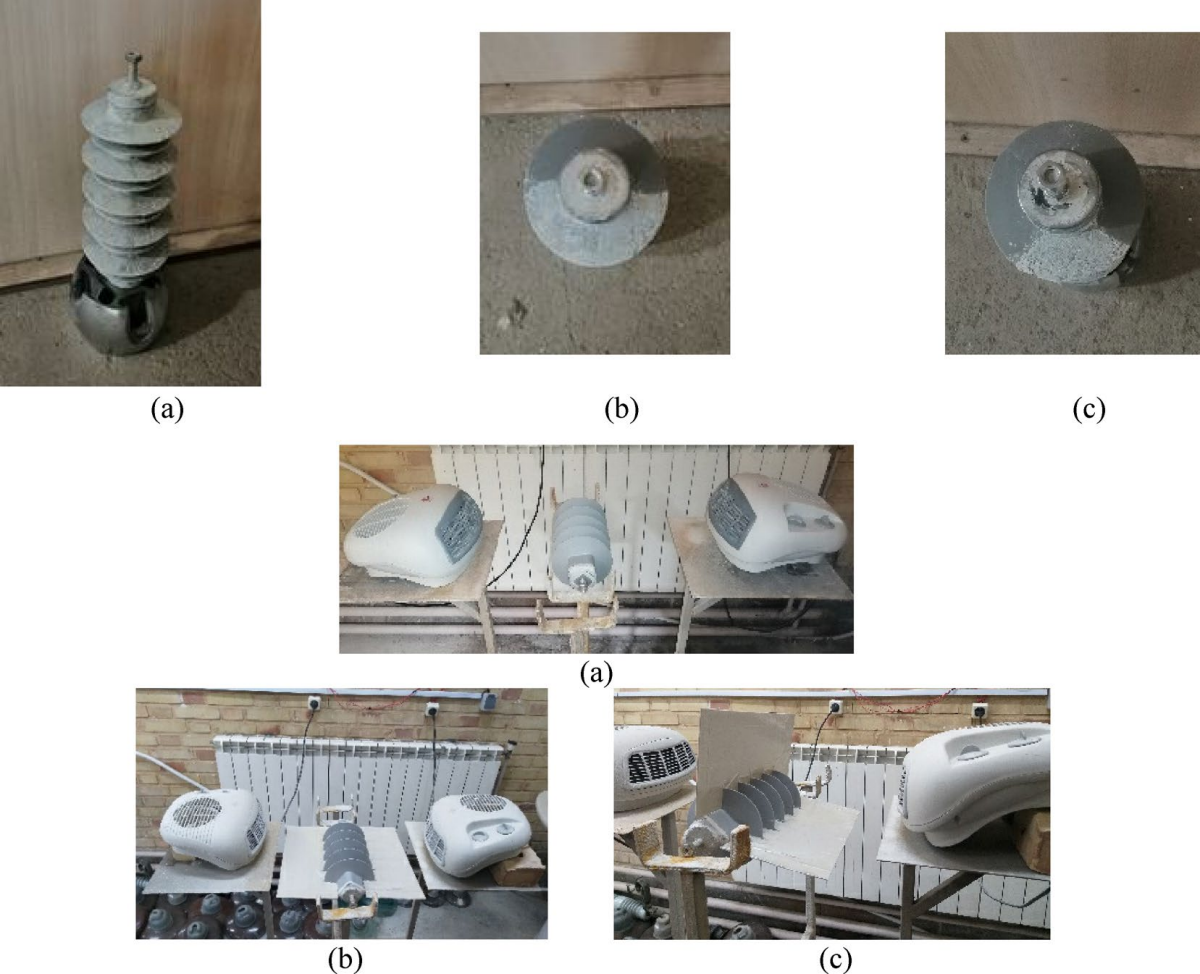
MOSA contamination set: uniform and non-uniform contamination on the MOSA (**a**) K = 1, (**b**) K = 0.5, (**c**) K = 0.25.

Due to the hydrophobic nature of silicon rubber housing of MOSAs, solid layer method as a suitable technique according to IEC 60,507 standard has been used^20^. In order to achieve the required electrical conductivity, the solution obtained from kaolin, salt (NaCl) and distilled water according to the values presented in Table 2 is sprayed on the surface of the MOSA^16^. Also, to accelerate the MOSA contamination process, two heaters with a certain distance from MOSA have been used. In order to create non-uniform fan-shaped contamination, by using two plates, the part of the MOSA that needs to be contaminated will be separated from the other parts. In this article, two angles of 90 and 180 degrees are considered for installing plates to produce sector pollution. Figure 4 shows the pollution collection of MOSA based on the above description.

**Table 2:** Amount of kaolin, salt and distilled water for different pollution levels.

Pollution level	SDD (mg/cm^2^)	Distilled water (l)	Salt (g/l)	Kaolin (g/l)
Very light	0–0.03	1	10	40
Light	0.03–0.06	1	20	40
Medium	0.06–0.1	1	40	40

By measuring the electrical conductivity of the pollution solution with a conductivity meter, the density of the salt deposit can be calculated. ESDD was calculated based on IEC 60,507 standards in order to calculate the pollution intensity of the MOSA surface using the following equations^21^:

1 $${\text{ESDD }} = \, \left( {{\mathrm{S}}_{{\mathrm{a}}} \times {\text{ V}}} \right)/{\text{ A}} \left( {{\mathrm{mg}}/{\mathrm{cm}}^{{2}} } \right)$$

2 $${\text{Sa }} = \, \left( {0.{57 }\sigma_{{{2}0}} } \right){ 1}.0{3 }\left( {{\mathrm{mg}}/{\mathrm{cm3}}} \right)$$

3 $$\sigma_{20} = \sigma_{\theta } (1{-}b({-}20)) \left( {S/cm} \right)$$

where Sa is the salinity and V is the volume of Contaminated solution, A is the cleaned surface area (cm2), σ_20_ is the electrical conductivity at 20 degrees Celsius (S/m), σ_θ_ is the electrical conductivity at θ temperature, and b is the temperature-dependent coefficient. For introducing the non-uniform pollution, the K parameter is defined for degree of contamination using Eq. (4). According to this parameter, K is equal to 1, 0.75, 0.5, and 0, respectively, for the clean state, non-uniform contamination of 90 degrees, non-uniform contamination of 180 degrees, and complete uniform contamination.

4 $$\mathrm{K}=\frac{\mathrm{CA}}{\mathrm{TA}}$$

where, CA is the clean cross section and TA is the total cross section of the conductor.

### Measuring the leakage current of the surge arrester

**Fig. 5:**
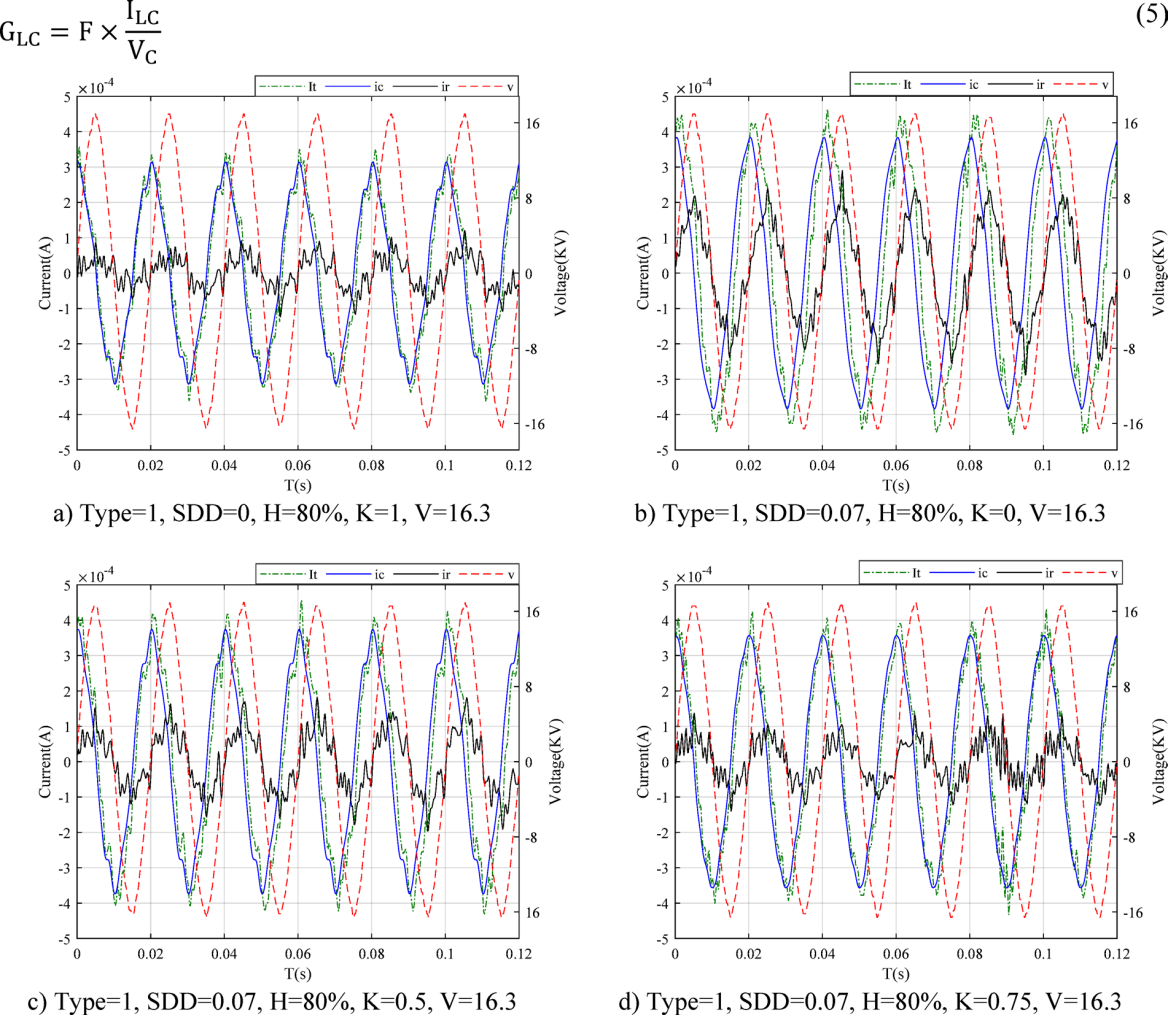
Leakage current waveforms for the MOSA type 1 at voltage of 16.3 kV.

One of the first and most important requirements in this paper is the correct and accurate measurement of the leakage current signal. The leakage current and voltage signal are measured and saved based on the described laboratory setup. Since the total leakage current includes two main components, separation of capacitive and resistive components is done based on the orthogonal current method^22^. In this article, the leakage current in clean state and three levels of very light, light and medium pollution under dry conditions and humidity of 50, 60, 70, 80 and 90% have been measured for uniform and fan-shaped type non-uniform pollution of 90 and 180 degrees. Figure 5 shows the leakage current of MOSA for type 1 at different values of K and certain pollution and humidity. The total leakage current in Fig. 5a is very similar to the capacitive component because it is extracted in the clean state. In fact, the effect of the resistive component in this case is insignificant and the total current follows the capacitive component. Figure 5b, c and d show that with the increase in the pollution level, the effect of the total leakage current resistive component has increased. Also, this factor has reduced the phase between the total leakage current and the resistance component.

### Measuring the electrical conductivity of the contaminated layer

**Fig. 6:**
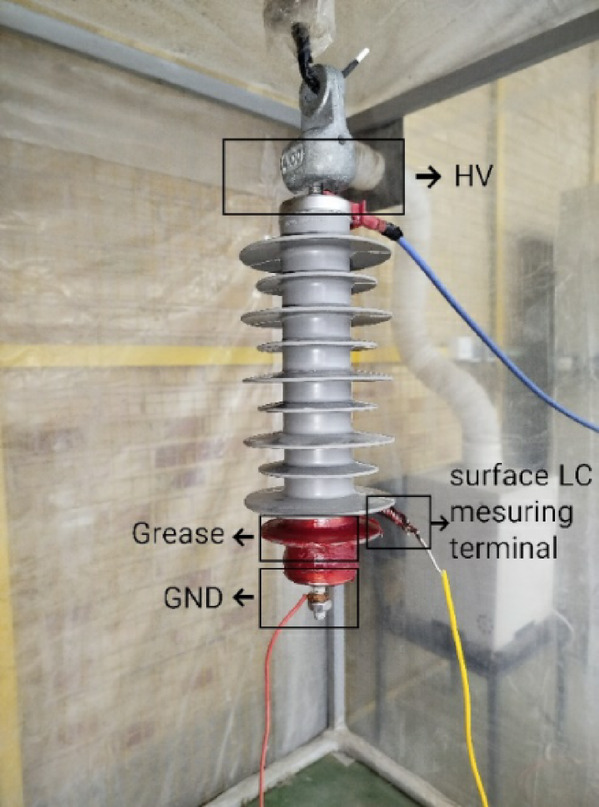
Separation of the internal and external current of the surge arrester.

As mentioned in the previous section, contamination has a great impact on the total leakage current. Meanwhile, the current passing through the varistors also affects on this current. In order to investigate the effect of contaminated layer and to obtain electrical conductivity information of its, it is necessary to separate the current passing through the surface and varistors. Reference^23^ has presented a suitable method for separating two components. According to this method, a metal fastener is used to connect the surface leakage current measuring probe near the MOSA ground terminals. Grease is used in the below part the metal fastener to disconnect surface leakage current from internal current. Figure 6 shows the requirements for measuring the surface leakage current in order to calculate the electrical conductivity of the contaminated layer. By placing the MOSAs in the laboratory test chamber, the surface leakage current was measured for all defined laboratory conditions. According to the IEC 60,815 standard, it is necessary to pay attention to the fact that the voltage applied to measure the surface leakage current should be sufficient to prevent a partial discharge on the surface of the surge arrester and the pollution layer. Based on this, in order to prevent the formation of dry band, leakage current measurement was performed at voltage levels of 0.7, 1, 1.5 and 2 kV. Equation (5) is used to calculate the electrical conductivity of the contamination layer^24^.

5 $${\mathrm{G}}_{\mathrm{LC}}=\mathrm{F}\times \frac{{\mathrm{I}}_{\mathrm{LC}}}{{\mathrm{V}}_{\mathrm{C}}}$$

where G_LC_ is the electrical conductivity, I_LC_ is the leakage current (mA), Vc is the critical voltage (kV) and F is the geometric form factor of the insulation, which is determined from Eq. (6)^24^:

6 $$\mathrm{F}={\int }_{0}^{L}\frac{l}{2\pi r(s)}ds$$

The term *2πr(s)* in Eq. (6) represents the perimeter of the insulating surface at a distance *l* along the creep path *L*.

**Fig. 7:**
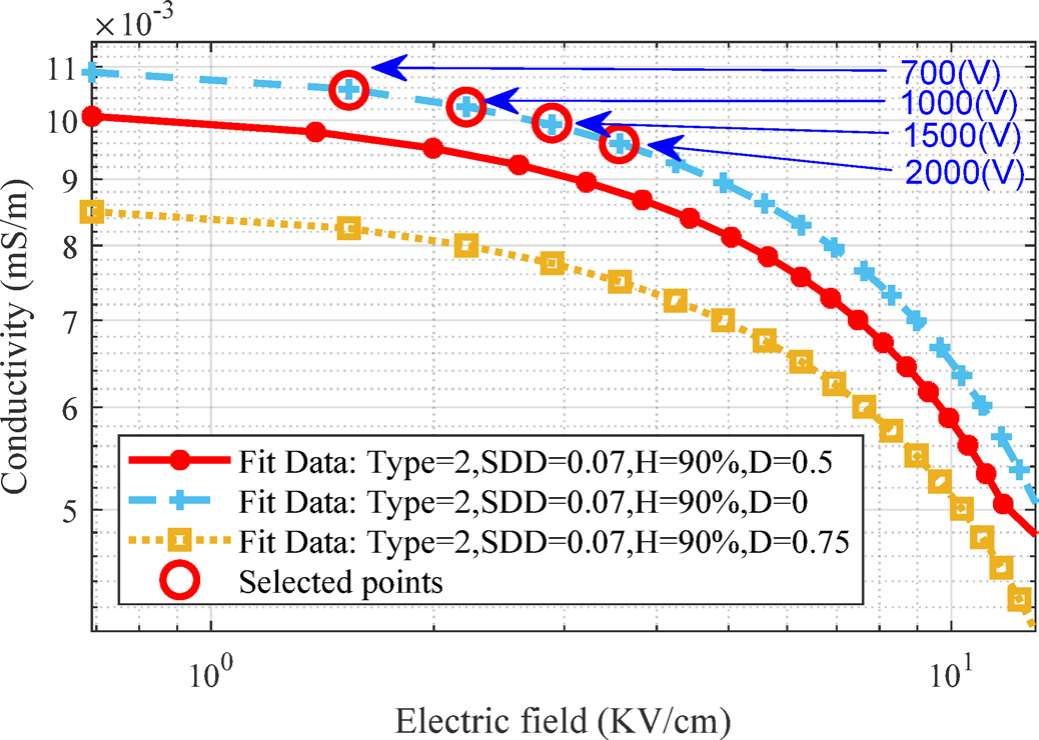
Changes in electric conductivity according to the electric field in different K conditions.

Using the measured surface leakage current per defined voltage, it is possible to fit the electrical conductivity curve of the contamination layer for a wider range of electric field intensity. Figure 7 shows the real and fitted data of pollution layer electrical conductivity for medium pollution, 90% humidity and three states of D versus to the electric field. As can be seen, the electrical conductivity has been calculated at four voltage levels of 0.7, 1, 1.5 and 2 kV to prevent surface discharge.

## Estimation of electrical conductivity

As mentioned, the current passing through the surface of the MOSA is very variable due to the influence of environmental conditions and is completely dependent on the electrical conductivity of the MOSA surface. Based on this, focusing on the modeling of the electrical conductivity of the contaminated layer was investigated as an important objective. In order to achieve a high-accuracy model that has the ability to consider all variables including humidity, pollution level, pollution type and voltage level, four methods LSTM, Random Forest, Linear Regression, CNN have been used in the Python environment.

With the aim of modeling the electrical conductivity of the contaminated layer, several scenarios were considered for conducting laboratory tests. These scenarios were defined based on the change of surge arrester type, SDD, K and H.

To apply methods based on artificial intelligence, it is necessary to consider part of the data for training the artificial network and another part for testing. In this paper, 70% of the data is used to train the model and the remaining 30% is used to evaluate the model’s performance. This partitioning is usually useful to avoid overfitting, as it can evaluate the performance of the model on previously unseen data.

In order to evaluate the performance of the artificial intelligence model and compare the methods used, two parameters Mean Squared Error (MSE) and Coefficient of Determination (R^2^) have been used. MSE is usually used to evaluate the accuracy of a regression model. MSE indicates the mean square of the model’s prediction errors. R^2^ criterion is also used to evaluate the performance of a regression model. R^2^ shows the degree of agreement between the actual and predicted values by the model. In addition, two loss and accuracy curves have been extracted for each of the methods, which show the performance of the model during the training and evaluation process. These curves provide necessary information about how the error and accuracy of the model changes during 2000 iterations. The loss curve shows the model error for each iteration. While the accuracy curve shows how well the training and validation data are correctly classified by the model. Examining these curves can help to identify problems such as overfitting or underfitting, and it creates the ability to adopt different strategies to improve the performance of the model.

By carefully analyzing these curves, it is possible to modify the training parameters, model settings and optimization methods to improve the overall efficiency of the model. For example, if the loss curve in the validation data is very high compared to the training data, the model may be overfitting and we need to adjust different parameters or use regularization techniques.

### Linear regression

Linear regression is one of the simplest and most widely used machine learning methods in the field of data analysis and statistical modeling. This method is used to predict a dependent variable (output) based on one or more independent variables (input). The main goal in linear regression is to find a linear relationship between the independent and the dependent variables. One of the features of this model is its simplicity and comprehensibility, fast and low-cost calculations, and easy interpretation of model coefficients. However, considering a linear relationship between variables, sensitivity to outliers and the inability to model non-linear conditions can be considered as its weaknesses.

**Fig. 8:**
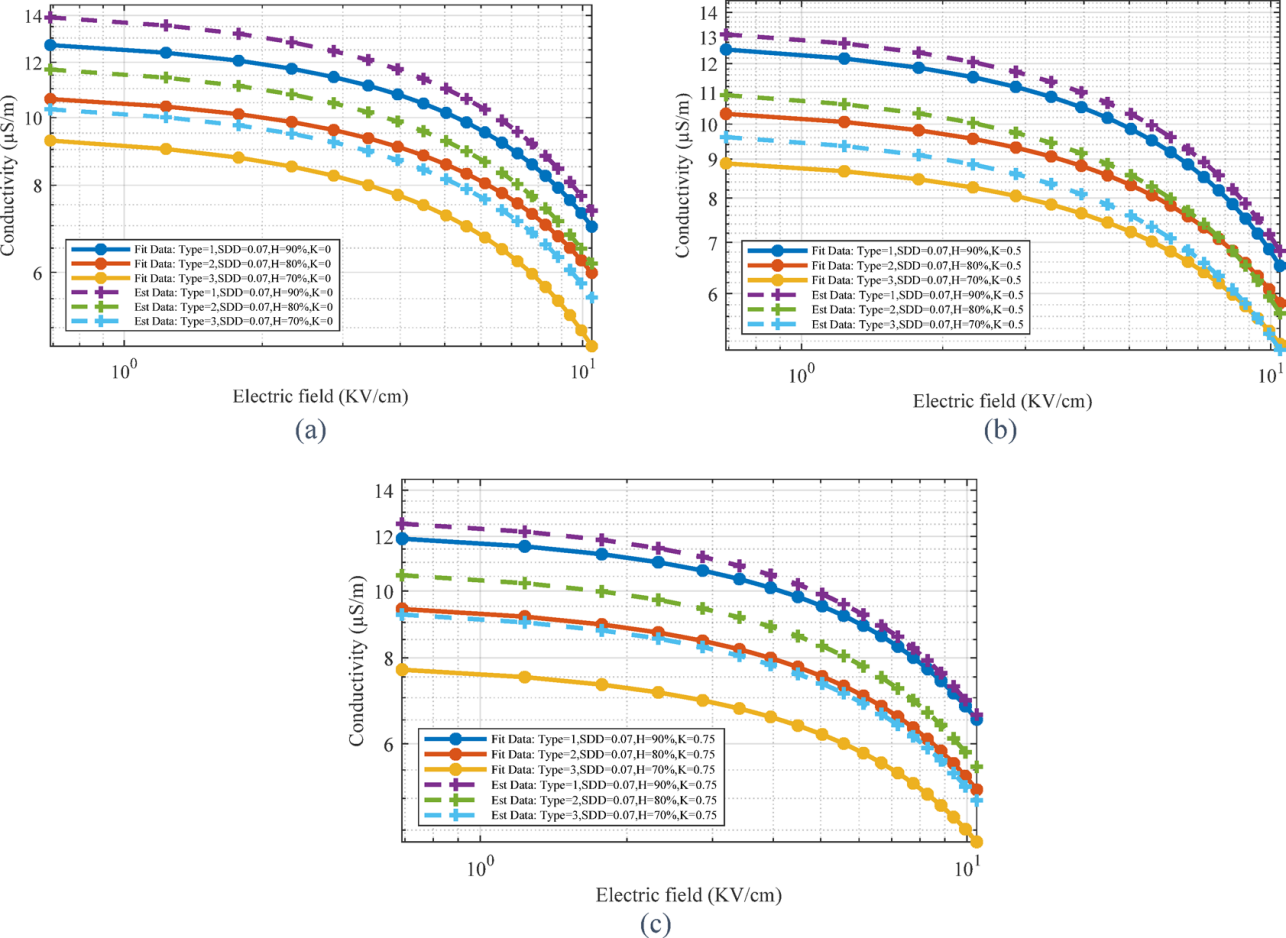
Estimation curve obtained from the linear regression method for real data.

After training the linear regression model, validation and testing steps will be performed using the new data to check the performance of the extracted model. Based on the results obtained in the linear regression model, the MSE is equal to 0.0105 and the R^2^ value is equal to 0.9959, which shows that the model has been able to predict more than 99% of the changes in the data. Figure 8 shows the comparison of the results obtained from the estimation of electrical conductivity using the linear regression method with the results obtained from the laboratory test.

**Fig. 9:**
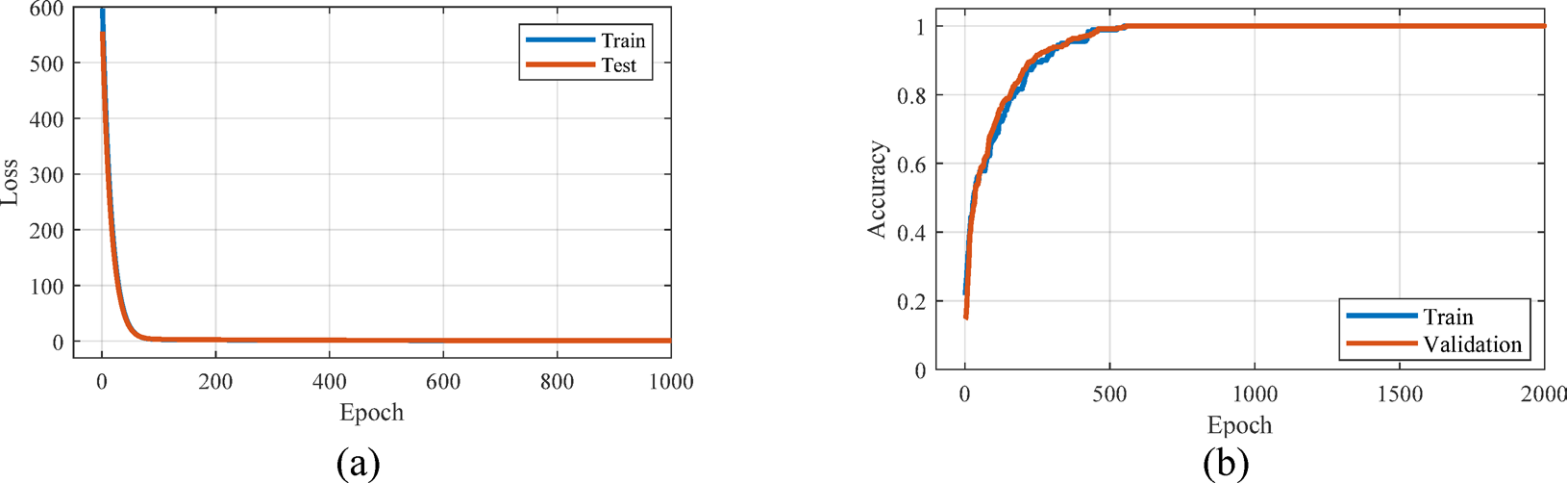
Curve of accuracy and loss obtained from linear regression method for training and validation data.

In Fig. 9, accuracy and loss curve for training and validation data set for linear regression method is shown. As can be seen, the loss curve for both data sets gradually decreased and reached a value of 0.98 in 814 iterations, which indicates the effective learning of the model. Additionally, the accuracy curve for both data sets progressively increased and reached a value of 1 by the 550th iteration, indicating an improvement in the model’s prediction accuracy. These results indicate that the model has successfully learned and can effectively predict new data.

### LSTM

LSTM (Long-Short Term Memory) neural network is a special type of recurrent neural network (RNN) designed for sequential data and time series. In these networks, information is passed from the previous stage to the next stage, so the network can remember the previous information and use it for future predictions. LSTM is developed to solve the vanishing gradient problem in traditional RNNs. These networks are able to learn long-term dependencies in ordinal data. By controlling the residual information, the input data and finally the output information, LSTM passes the important information network along the sequence chain to obtain the desired output. In this process, using the optimization method, the weights of the model are updated to minimize the prediction error. However, LSTM is sensitive to training data and the quality and quantity of input data, which has a direct impact on the final performance of the model, so that insufficient or inappropriate data can lead to poor performance.

**Fig. 10:**
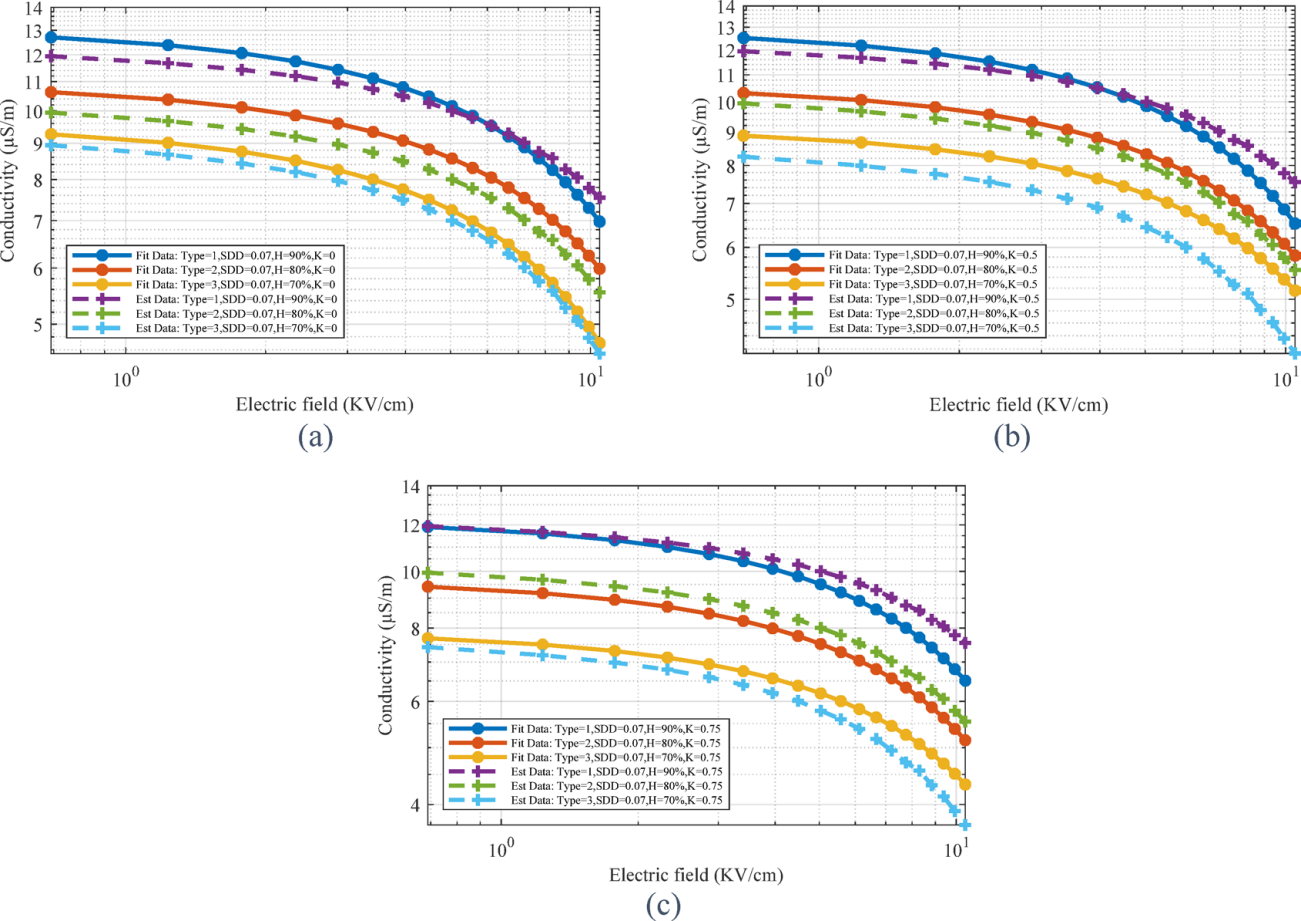
Estimation curve obtained by LSTM method for real data.

**Fig. 11:**
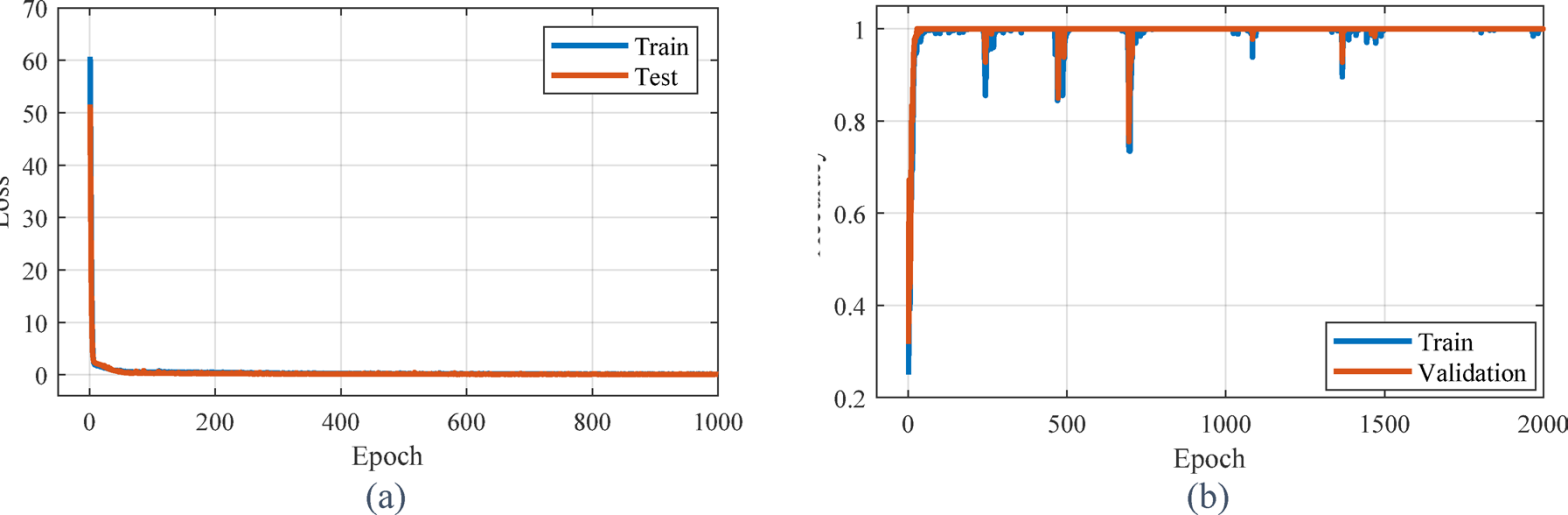
Accuracy and loss curve obtained from LSTM method for training and validation data.

The comparison of the results obtained from the estimation by LSTM method and the laboratory results is shown in Fig. 10. In order to quantitatively evaluate the LSTM method, MSE and R^2^ parameters were calculated. The MSE and R^2^ values obtained for the LSTM model show that the performance of this model is slightly weaker than the linear regression model. The mean square error of the presented LSTM model is equal to 0.333. Also, the value of R^2^ is equal to 0.841. According to the R^2^ value obtained, it can be concluded that the LSTM model does not have sufficient ability to model and predict data changes. The results obtained from the loss and accuracy curves are shown in Fig. 11. As can be seen in Fig. 11a, with the increase in the number of iterations, the value of loss decreases and tends to zero. Figure 11b shows the prediction accuracy of the model versus iterations. Changes in the accuracy curve are observed, which are due to overfitting occurring in the training process. Meanwhile, in the above epochs, the accuracy characteristic has reached almost one, which shows the increase in accuracy in the classification of training and validation data. Although the loss curve reached its lowest value in under 1,000 iterations, the training process continued for up to 2,000 iterations. This extended training was necessary to achieve adequate accuracy.

### Random forest

Random forest is a machine learning algorithm used for classification and regression. This algorithm uses the combination of several decision trees in order to improve accuracy and prevent overfitting. The working method of random forest is such that the training data is randomly sampled and a different data set is created for each decision tree. Random forest has high stability and accuracy due to the use of several decision trees. Due to the use of averaging the results of several trees, the random forest is more resistant to overfitting. This algorithm can work well with large and complex data. The importance of features for prediction, which can be useful in data analysis, can be investigated by this method. Training and prediction with random forest may take more time than with simpler models. Due to the use of a large number of decision trees, the interpretation of the results in this method may be more difficult. Also, choosing the right number of trees and other parameters may require several adjustments and experiments.

**Fig. 12:**
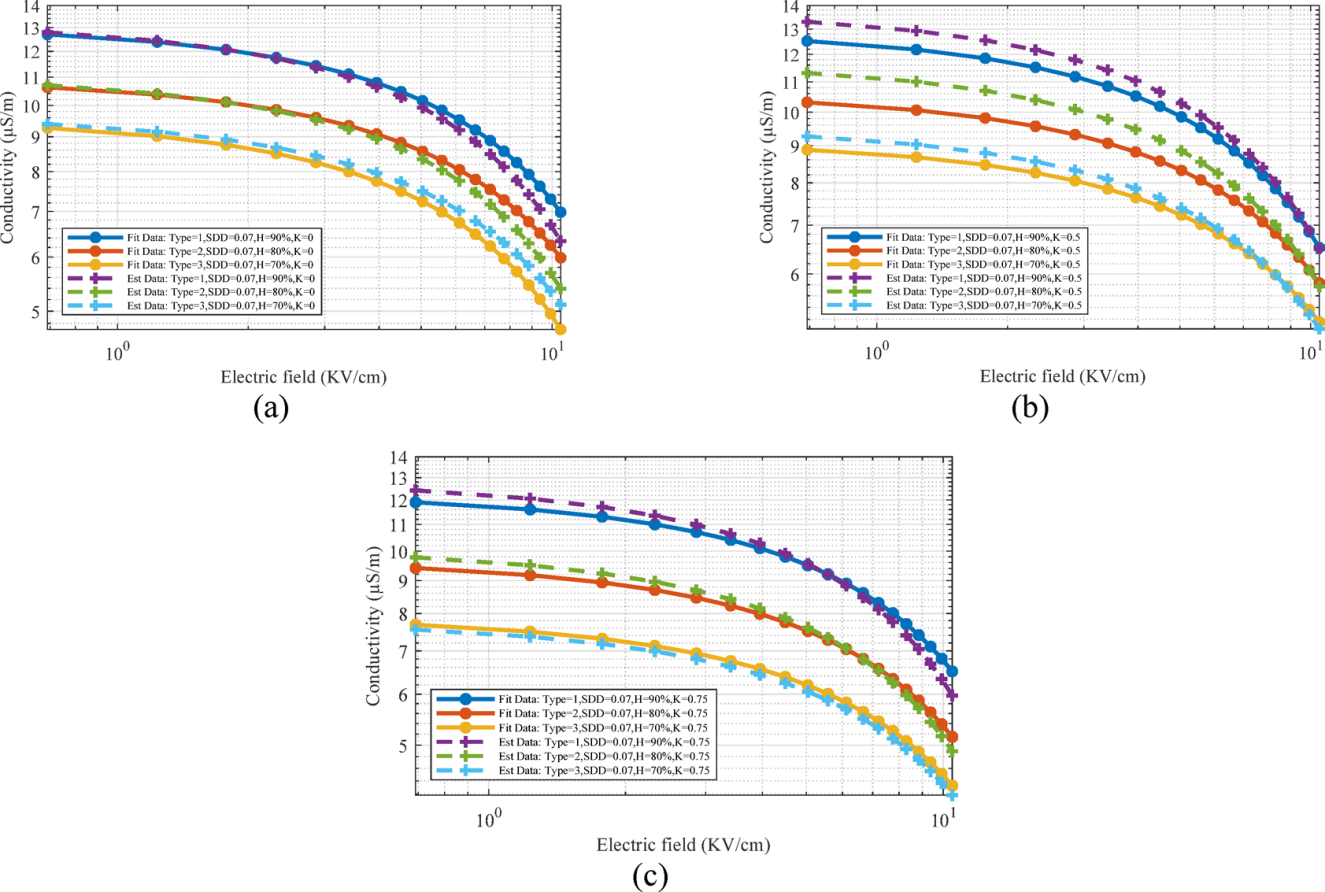
Estimation curve obtained from random forest method for real data.

**Fig. 13:**
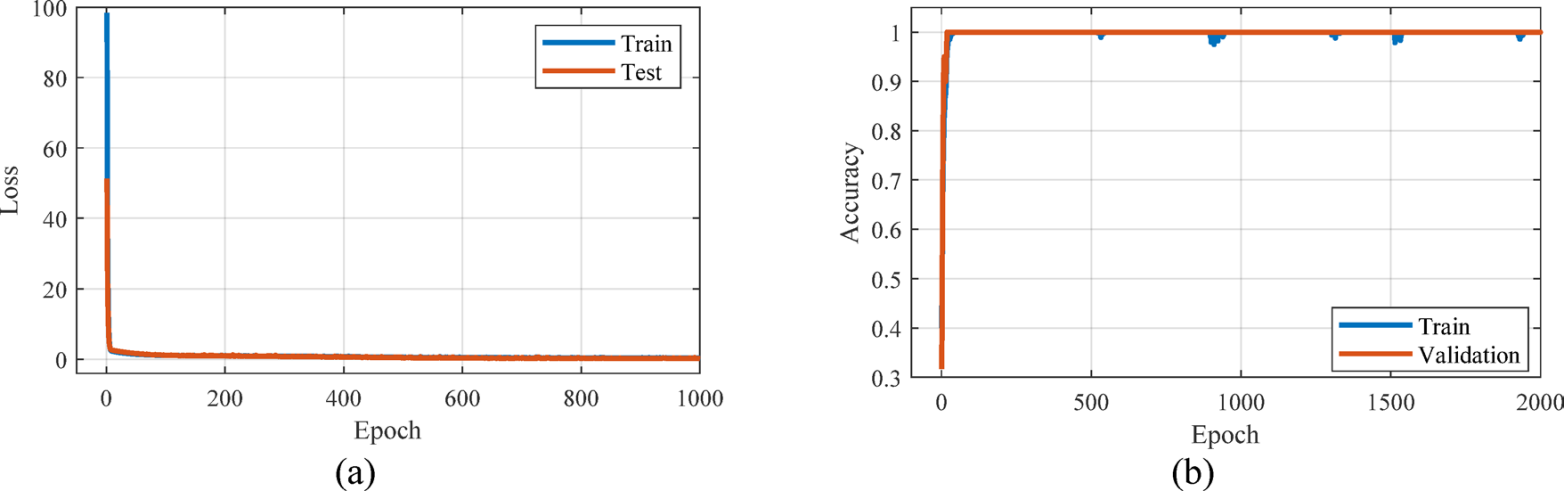
Accuracy and loss curves obtained from random forest method for training and validation data.

The mean square error using the random forest method is 0.000216, which is much lower than the two investigated methods. This shows that the presented model accurately predicts the data. The R^2^ value is 0.9999. Therefore, the presented model has the most conformity with real data and has the ability to interpret data changes by more than 99%. According to values of MSE and R^2^ and Fig. 12, it can be concluded that the proposed random forest model performed very well and provided a very good estimate of the data. The curve of loss and accuracy for the random forest method is shown in Fig. 13. As can be seen, the losses and accuracy of this method have reached the desired values, very quickly.

### CNN

As one of the most advanced and effective methods in the field of deep learning, CNN is used in many real applications due to its automatic feature extraction, suitable for multidimensional data, and high generalizability. This method is scalable and resistant to changing the size of the input, so it can be used for data with different sizes and dimensions without the need for large changes in the network structure. This method usually performs well in cases where the data has patterns or complex characteristics and non-linear dependencies. This is despite the possibility of over-fitting due to insufficient or insufficient training data. Choosing layers, sizes, and their sequence can be a complicated and time-consuming process.

**Fig. 14:**
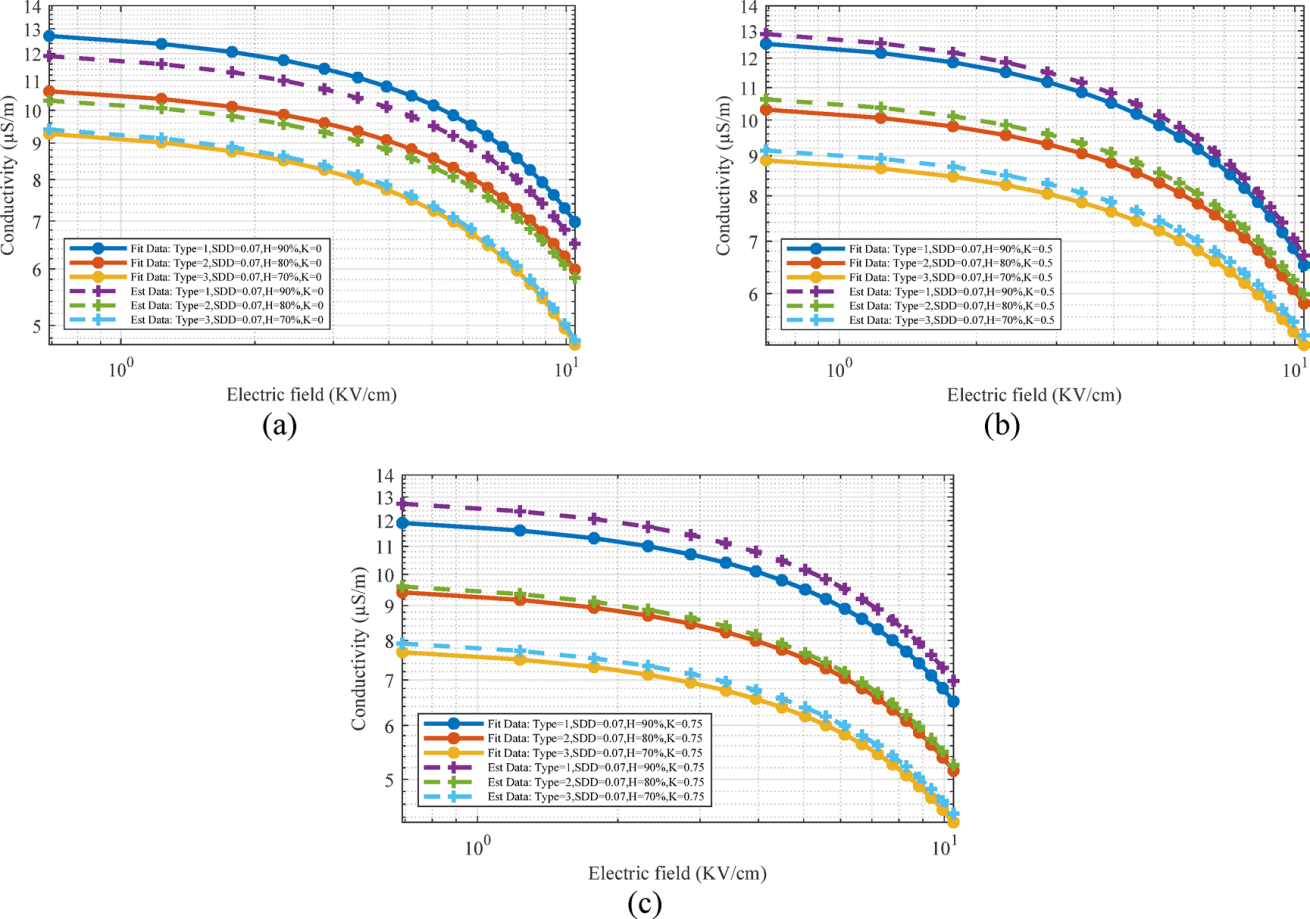
Estimation curve obtained from CNN method for real data.

**Fig. 15:**
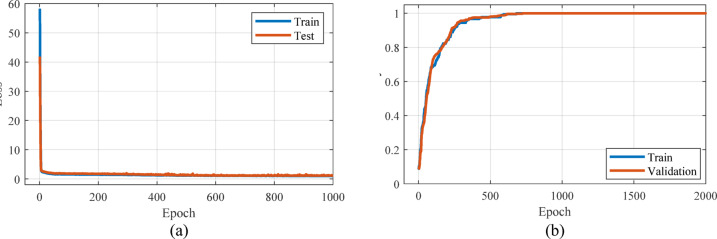
Accuracy and loss curve obtained from CNN method for training and validation data.

Figure 14 presents the comparison between the predicted electrical conductivity values using the CNN method and the experimental results from laboratory tests. MSE in this method is equal to 0.009587, which is in the optimal range. The value of R^2^ is also equal to 0.9962, which shows that this model has been able to have the best possible match with the real data and explain more than 99% of the changes in the data. The loss and accuracy results in Fig. 15 show that the CNN model is well trained and can predict new data with high accuracy. The stability of accuracy in both training and validation datasets indicates the proper performance of this method in preventing overfitting.

With the aim of achieving the best method for estimating the electrical conductivity of the pollution layer, the results of 4 methods are shown in Table 3. As can be seen, the random forest method with the lowest MSE value and the highest R^2^ value is considered as the greatest estimation method. The time comparison of the methods shows the high speed of the linear regression method in estimation. Although the R^2^ value in this method is acceptable, the MSE value is about 48% higher than the random forest method, which shows the weakness of this method.

**Table 3:** MSE and R^2^ values for different methods.

Method	Linear regression	Random forest	LSTM	CNN
MSE	0.010539	0.000215	0.333360	0.009587
R^2^	0.995968	0.999917	0.841321	0.996274
Time running(s)	2.638815	7.005303	7.775573	2.978743

**Fig. 16:**
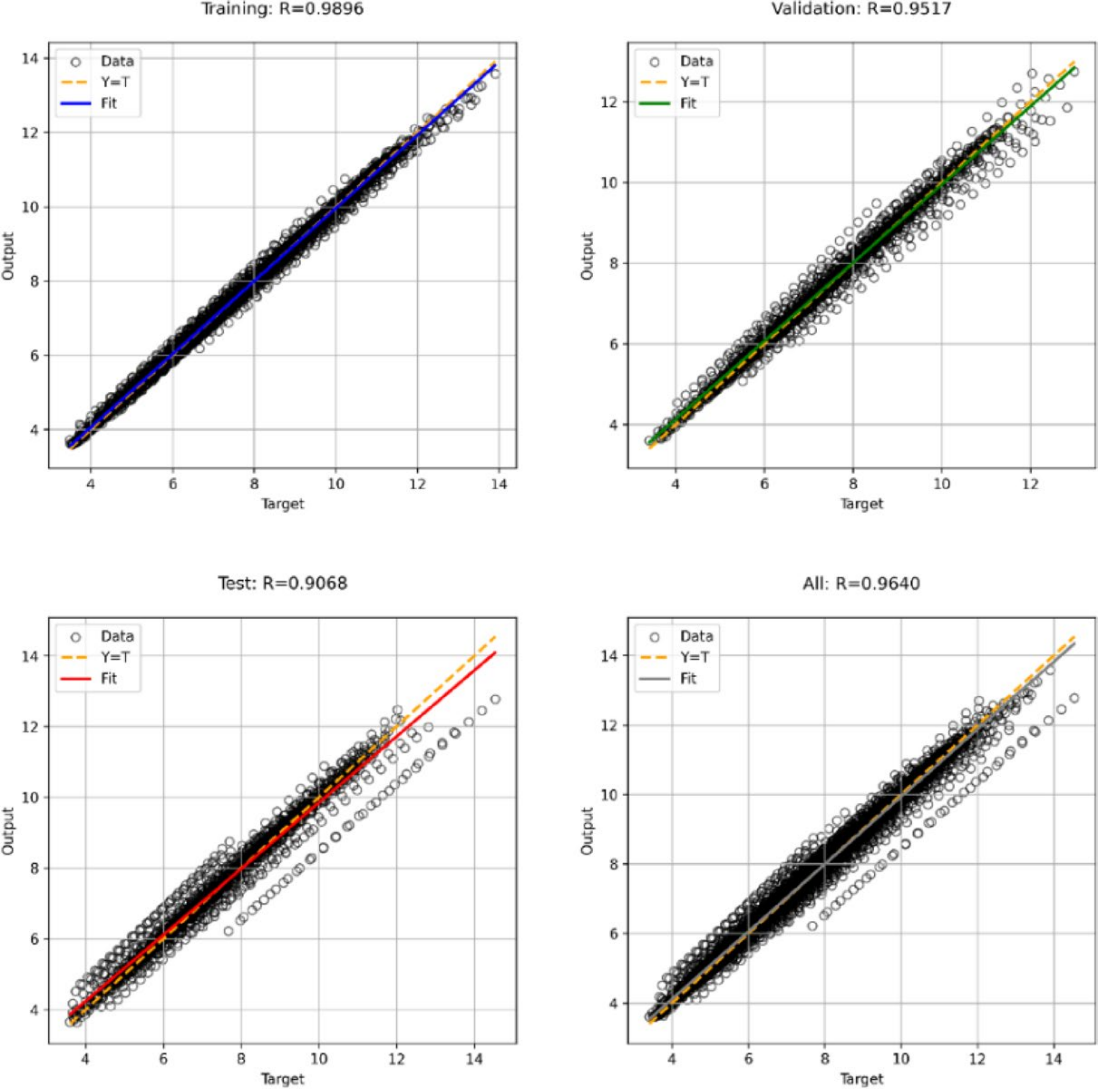
Regression charts for random forest method.

Figure 16 shows the performance scatter diagram of the presented model for the random forest method in different sets of data. Each graph contains predicted values (Output) in terms of actual values (Target). The determination coefficient R is also calculated for each chart. The coefficient of determination of R^2^ for the training data is equal to 0.9896, which, according to the fitting and reference lines, indicates a very strong fit between the estimated and actual values. The value of R coefficient for validation and test data is equal to 0.9517 and 0.9068, respectively, which are lower than the training data. In fact, due to the reduction of the data number used in this section, the probability of estimation error has increased and the value of R has decreased. Finally, the value of R for all data was presented, which is equal to 0.9640. A small difference between R in different data sets shows that the model is well adapted and overfitting is avoided.

## Finite element method-based modeling process

The finite element method is a numerical technique used to solve partial differential equations (PDEs) in order to represent and analyze physical systems. Using software based on this method, electric field, electric potential and LC analysis has been performed. With the aim of considering the effect of fan-shaped pollution, the MOSA model was simulated in a three-dimensional environment. To model the surge arrester, the first step is to draw its geometry. In the next step, the necessary parameters are determined according to the materials of each part of the surge arrester loaded in the simulation and the boundary conditions. The characteristics of the materials used in model are given in Table 4.

**Table 4:** Electrical conductivity value and relative permeability for different parts of the surge arrester.

Material	Relative permittivity, εr	Electrical conductivity, σ (S/m)
Air	1	1e-29[S/m]
Silicon rubber	11.7	1e-12[S/m]
Aluminum	1	3.030e7[S/m]
Pollution layer	80	According to the estimated values obtained from the proposed model
Zno	10.8	According to V-I characteristic of MOSA

**Fig. 17:**
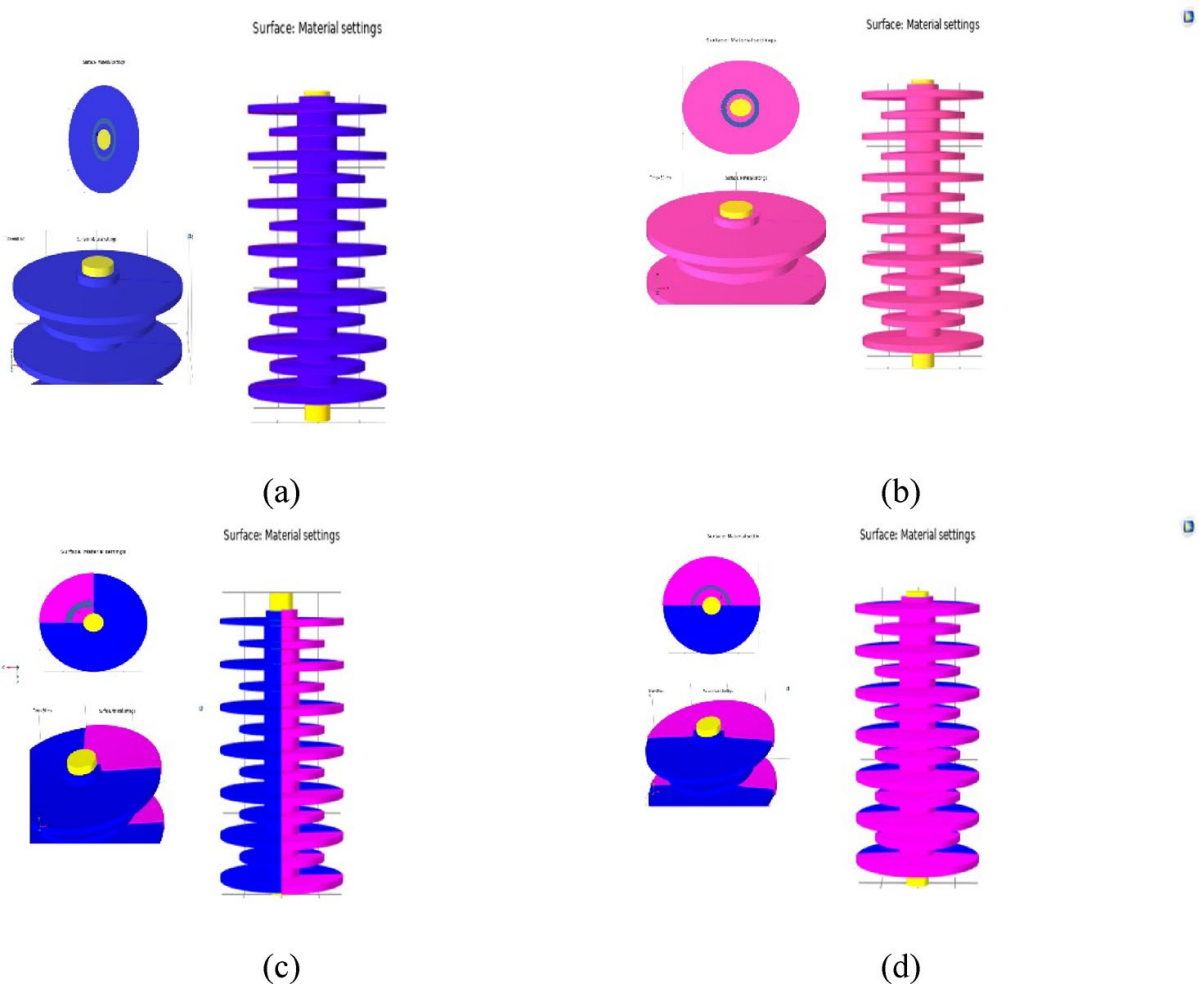
different surge arrester arrangements in clean condition (**a**), uniform contamination (**b**) and 90 (**c**) and 180-degree (**d**) sectors.

Figure 17 shows the simulated surge arrester for clean condition, uniform and fan-shaped type pollution. In order to model the pollution layer, a 1 mm thick layer is considered on the surface of silicone rubber.

**Fig. 18:**
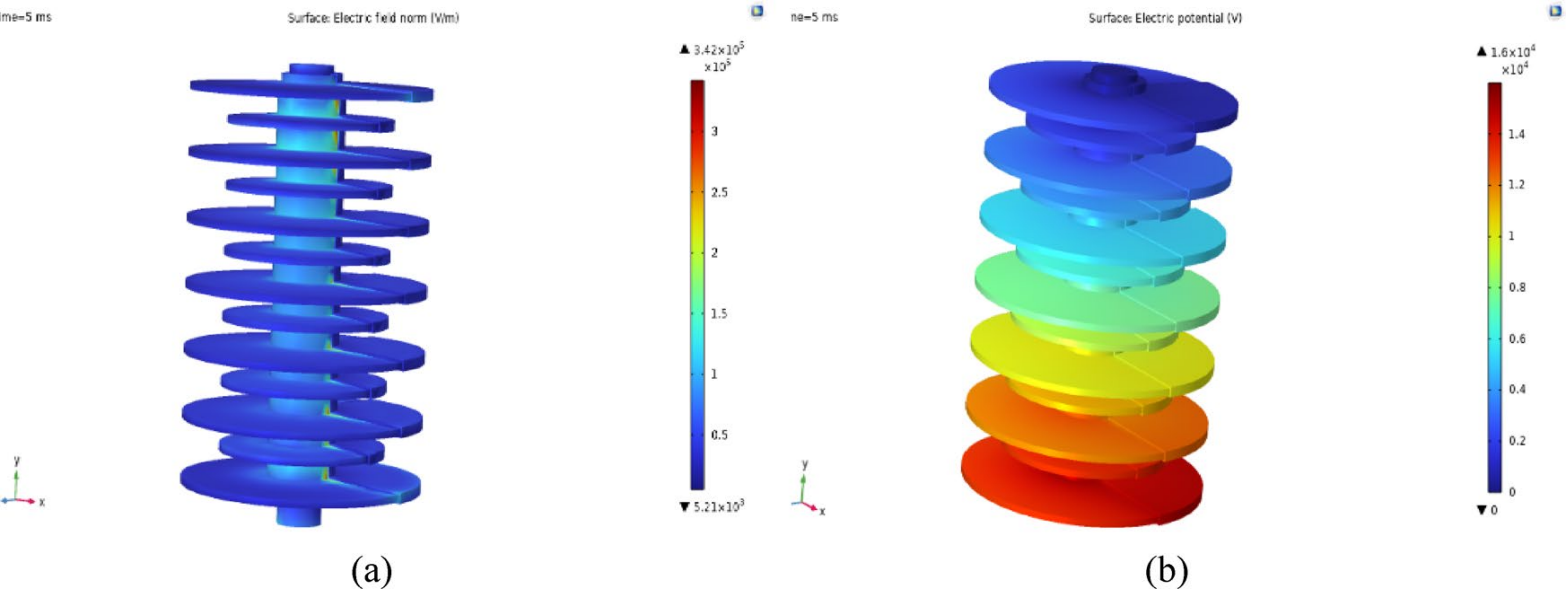
Changes of the electric field and electric potential along the line passing through the surge arrester in the finite element software environment.

Potential and electric field distribution along the line passing through the MOSA housing for K = 0.75 are shown in Fig. 18. By applying a voltage of 16.3 kV to the upper flange of the surge arrester and grounding its lower flange, it is possible to evaluate the changes of the field and electric potential along the longitudinal direction of the surge arrester and along the creepage distance.

**Fig. 19:**
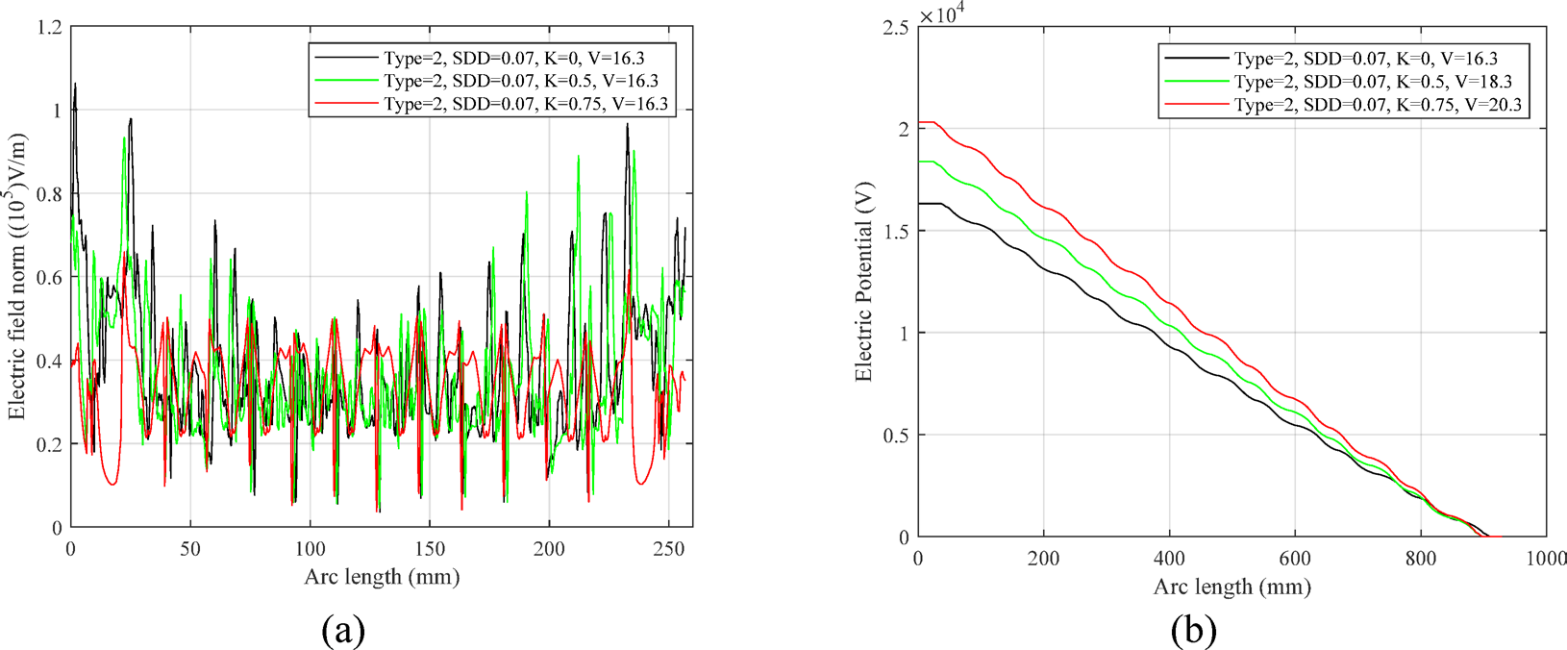
Changes of the electric field and electric potential along the line passing through the surge arrester.

The diagram in Fig. 19a shows the distribution of the field and Fig. 19b shows electric potential according to the creepage length of the surge arrester for uniform and non-uniform pollution. In the field distribution diagram, significant changes can be seen at the edges of the shed, which indicates the intensification of the electric field in those places. For showing the electric field, the type of MOSA and the parameters such as SDD and V are fixed, but the value of K changes. As can be seen, with the increase of K, the maximum intensity of the electric field has decreased due to the boundary conditions between the contaminated layer and the clean area of the surge arrester surface. Figure 19b shows the changes in the potential distribution of the MOSA according to the voltage amplitude, which decrease with the decrement in voltage level and increment of the distance from the high voltage flange.

Considering that the purpose of simulation in the FEM based software environment is to achieve the leakage current of MOSA, it is necessary to evaluate the current modeling process. Current variations in FEM simulation follow Eq. (10)^13^.

10 $$J = \sigma E + Je$$

where σ represents the electrical conductivity in S/m and Je represents the generated external current density in A/m2. According to the equation E =—∇V and considering the divergence in Maxwell Ampere’s law^21^:

11 $$\nabla \cdot (\nabla \times H) = \nabla \cdot \left[J+\frac{\partial D}{\partial t}\right]=0$$

where J represents the current density and *∂D/∂t* represents the displacement current density. By numerically solving this equation in the time domain, the electric field and electric potential distribution can be obtained. This can be expressed as follows^21^:

12 $$-\nabla \cdot \frac{\partial \left(\varepsilon r \varepsilon \nabla V\right)}{\partial t}- \nabla \cdot (\sigma \nabla V)=0$$

Here, σ represents the electrical conductivity, εr represents the relative permittivity, and V represents the electric potential.

## Results and discussion

In order to analyze and evaluate the performance of the created model for simulating the MOSA leakage current based on the estimation of the electrical conductivity of the contaminated layer, three scenarios have been defined.

The first scenario S1: The purpose of S1 is to validate the proposed model based on electrical conductivity data, known as fitted data. In this scenario, the leakage current is simulated based on the electrical conductivity values which have been obtained from laboratory tests at different conditions.

The second scenario S2: The purpose of this scenario is to simulate the leakage current from the FEM model based on electrical conductivity data, known as estimated data by the Random Forest method. The input conditions for estimating the electrical conductivity characteristic are the same conditions used to train the AI-based model.

The third scenario S3: The purpose of this scenario is the same as the second scenario based on electrical conductivity data, known as predicted data by the Random Forest method. The difference between S2 and S3 is that the input conditions for the prediction of the electrical conductivity characteristic are not the same as the conditions used to train the AI-based model.

### First scenario evaluation

**Fig. 20:**
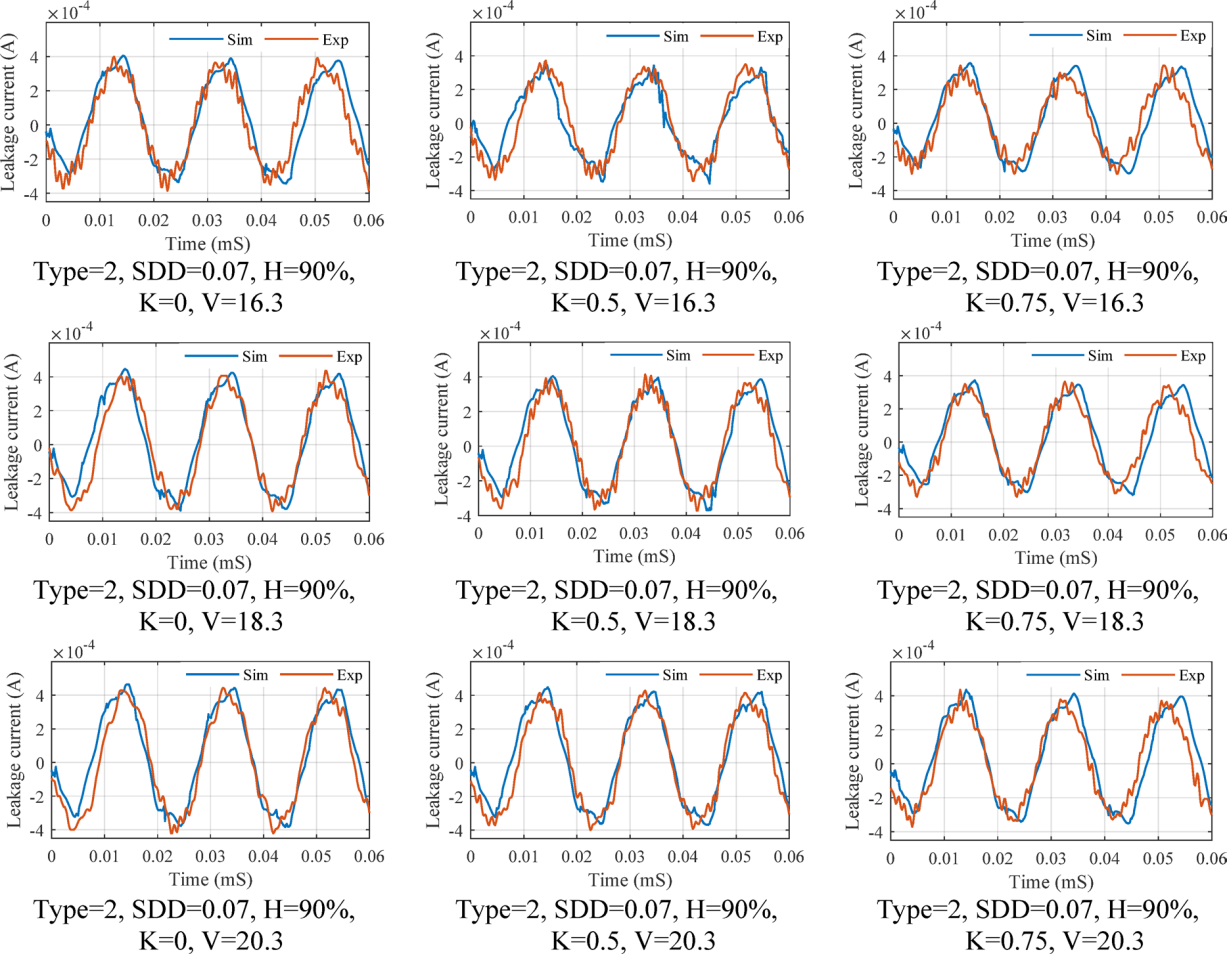
Comparison of the leakage current obtained from the laboratory test with the FEM simulation results.

The results extracted from the FEM model and the laboratory test for the leakage current of the surge arrester under fitted data of polluted layer conductivity, different conditions of pollution, environment and voltage for type 2 surge arrester are shown in Fig. 20.

**Fig. 21:**
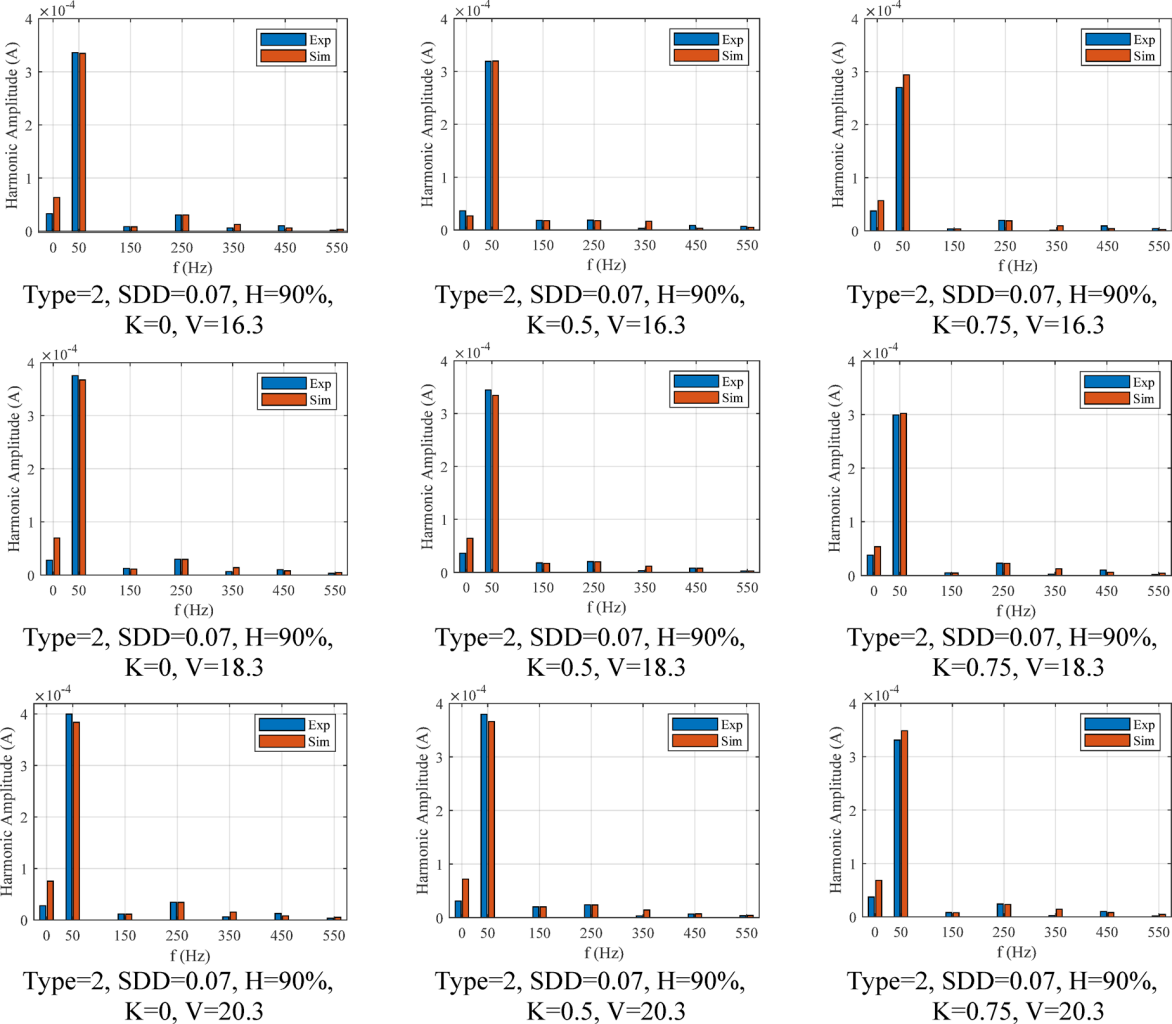
Comparison of leakage current harmonic component amplitude obtained from laboratory test with FFT of FEM simulation results.

From the appearance of the signals, it can be inferred that the leakage current amplitude has been simulated with very high accuracy by the FEM model according to the operation conditions. However, what is important in the field of evaluating the surge arrester performance is their harmonic components. Since the harmonic components have a decisive role in detecting the surge arrester condition, the first to eleven harmonic components corresponding to the leakage currents shown in this scenario are shown in Fig. 21. Nevertheless, since the first, third and fifth harmonic components of the total leakage current are more effective than the change of environmental conditions, a comparison of these harmonic components has been made. Table 5 shows the error percentage of harmonic components for the results presented in Fig. 21. As shown in this table, the highest error rate of the first, third, and fifth components is 8.82, 7.9, and 8.5%, respectively. The average value of error and standard deviation for all of the data of the leakage current is equal to 3.11% and 3.22, respectively, which is acceptable in simulating the MOSA leakage current.

**Table 5:** First, second and third harmonic error value for the type 2 surge arrester, three voltage level, humidity, three pollution level and three pollution type in percentage for first scenario.

Current Component			I_r1_			I_r3_			I_r5_		
Voltage and SDD		K	360	180	90	360	180	90	360	180	90
		Humidity %	Error (%)			Error (%)			Error (%)		
V_1_=16.3	SDD=0.015	70	3.1795	3.0043	5.5684	2.7309	7.8566	2.0201	1.1865	2.1317	6.8267
		80	8.0814	1.5823	6.0014	7.9053	0	1.9963	1.6375	8.301	3.6792
		90	7.2393	5.2814	0.6119	0	0	0	1.7282	1.4129	1.4734
	SDD=0.03	70	3.8354	3.3478	4.5463	2.5082	7.8341	1.8511	0.9470	3.5920	5.4816
		80	8.4128	1.7830	5.2511	7.9081	0	1.5106	1.6266	1.4294	1.3651
		90	6.5295	5.0277	8.036	0	0	0	1.7204	1.4017	1.3715
	SDD=0.07	70	3.3569	2.7091	4.8355	1.6994	7.821	1.9634	0.6161	3.5632	5.4696
		80	8.8382	1.1193	5.6479	7.5681	0	2.0382	1.6224	1.4193	1.3546
		90	6.9736	5.6063	8.354	0	0	0	1.7135	1.3919	1.3613
V_1_=18.3	SDD=0.015	70	1.0632	0.6834	7.2885	0.1045	4.4673	5.3578	2.7816	1.0841	7.7102
		80	7.934	0.7748	7.8892	0	0	4.1165	1.6176	8.5022	1.3484
		90	8.1119	7.4532	1.3562	0	0	0	1.7091	1.5850	1.3550
	SDD=0.03	70	3.5922	2.8744	4.5538	1.6953	5.7933	3.3899	0.2171	3.9330	5.0965
		80	8.6339	1.2180	5.4533	7.5334	0	1.8977	1.6206	1.6266	1.3525
		90	6.7720	5.4784	7.0748	0	0	0	1.7119	1.5901	1.3588
	SDD=0.07	70	3.0728	2.2492	4.8725	0.8904	5.8290	3.9120	0.1080	3.8589	5.1162
		80	8.0485	0.5690	5.8428	7.1771	0	2.4801	1.6134	1.6134	1.3426
		90	7.1715	6.0717	8.3598	0	0	0	1.7050	1.5770	1.3488
V_1_=20.3	SDD=0.015	70	0.7628	0.2529	7.3244	0.6928	7.6395	5.8315	2.0463	1.4451	7.3672
		80	3.2841	1.3629	8.0812	0	0	4.5278	1.6087	1.6046	1.3361
		90	8.3525	7.9195	1.5335	0	0	0	1.7006	1.5691	1.3426
	SDD=0.03	70	2.1782	1.2557	5.5954	0.2634	6.9724	4.8795	0.1430	3.4901	5.4225
		80	8.8207	0.4621	6.6435	0	0	3.3713	1.6040	1.5965	1.3297
		90	8	7.0236	0.0276	0	0	0	1.6960	1.5608	1.3361
	SDD=0.07	70	1.7333	0.7040	5.9248	1.0569	6.9809	5.5166	0.4270	3.4209	5.4393
		80	7.2803	1.0777	7.0367	0	0	3.9239	1.5970	1.5833	1.3197
		90	8.4098	7.5842	0.3834	0	0	0	1.6890	1.5476	1.3261

### Simulation of leakage current based on S2 and S3

In this section, the analysis and evaluation of the leakage current obtained from the FEM software is discussed based on the electrical conductivity characteristic presented in the second and third scenarios. In each part, the results obtained from the proposed model have been compared with the laboratory test results.

#### Second scenario evaluation

In validation of the second scenario, input parameters include voltage, humidity, SDD and K have been considered correspond to the conditions used in the laboratory test process. After estimating the electrical conductivity characteristic by the proposed model, the estimated values are applied to the FEM model. Tables 6 and 7 compares the results of leakage current obtained from laboratory tests and extracted from FEM based simulated model. The results obtained for the first, third and fifth harmonics are shown for three K, voltage and humidity levels.

**Table 6:** Values of the total leakage current and first harmonics for the type 2 surge arrester, three voltage level, humidity, three pollution level and three pollution type in percentage for second scenario.

Current component			I_t_						I_r1_					
Voltage and SDD		K	360		180		90		360		180		90	
		Humidity %	Exp	Sim-S2	Exp	Sim-S2	Exp	Sim-S2	Exp	Sim-S2	Exp	Sim-S2	Exp	Sim-S2
V_1_ = 16.3	SDD = 0.015	70	0.3964	0.4037	0.3871	0.3495	0.3689	0.3575	0.3561	0.3340	0.2580	0.2630	0.2503	0.2530
		80	0.4625	0.4470	0.4278	0.4048	0.4111	0.3729	0.3797	0.3670	0.3054	0.3340	0.2687	0.2647
		90	0.5044	0.4644	0.4668	0.4483	0.4352	0.4363	0.3882	0.3830	0.3114	0.3074	0.2546	0.2506
	SDD = 0.03	70	0.4012	0.4126	0.3917	0.3531	0.3733	0.3654	0.3592	0.3413	0.2603	0.2688	0.3020	0.2994
		80	0.4681	0.4568	0.4329	0.4137	0.4160	0.3811	0.3223	0.3183	0.3078	0.3038	0.2705	0.2665
		90	0.5105	0.4746	0.4724	0.4582	0.4404	0.4459	0.3208	0.3168	0.3139	0.3099	0.25574	0.25174
	SDD = 0.07	70	0.4055	0.4158	0.3960	0.3599	0.3774	0.3682	0.3620	0.3440	0.2624	0.2709	0.3035	0.3018
		80	0.4731	0.4604	0.4376	0.4169	0.4206	0.3841	0.3248	0.3208	0.3100	0.3060	0.2712	0.2672
		90	0.5160	0.4783	0.4775	0.4617	0.4452	0.4494	0.3232	0.3192	0.3161	0.3121	0.25678	0.25278
V_2_ = 18.3	SDD = 0.015	70	0.4083	0.4089	0.3987	0.3620	0.3800	0.3621	0.3638	0.3383	0.2638	0.2664	0.3045	0.2968
		80	0.4764	0.4528	0.4406	0.4101	0.4234	0.3817	0.3263	0.3223	0.3114	0.3383	0.2720	0.2680
		90	0.5195	0.4704	0.4808	0.4541	0.4483	0.4420	0.3247	0.3207	0.2776	0.2736	0.25744	0.25344
	SDD = 0.03	70	0.4065	0.4179	0.3970	0.3577	0.3783	0.3701	0.3626	0.3458	0.2629	0.2723	0.3039	0.3033
		80	0.4743	0.4628	0.4387	0.4191	0.4216	0.3861	0.3253	0.3213	0.2705	0.2665	0.2715	0.2675
		90	0.5172	0.4808	0.4787	0.4641	0.4463	0.4517	0.3238	0.3198	0.2767	0.2727	0.25701	0.25301
	SDD = 0.07	70	0.4109	0.4212	0.4013	0.3645	0.3824	0.3730	0.3655	0.3485	0.2651	0.2744	0.3054	0.3057
		80	0.4794	0.4664	0.4435	0.4224	0.4261	0.3891	0.3277	0.3237	0.2727	0.2687	0.2727	0.2687
		90	0.5229	0.4846	0.4839	0.4678	0.4511	0.4552	0.3262	0.3222	0.2790	0.2750	0.25806	0.25406
V_3_ = 20.3	SDD = 0.015	70	0.4137	0.4143	0.4040	0.3665	0.3850	0.3669	0.3673	0.3427	0.2664	0.2699	0.3064	0.3007
		80	0.4827	0.4587	0.4465	0.4154	0.4291	0.3867	0.3293	0.3253	0.2742	0.2702	0.2735	0.2695
		90	0.5264	0.4766	0.4872	0.4600	0.4542	0.4477	0.3277	0.3237	0.2804	0.2764	0.25873	0.25473
	SDD = 0.03	70	0.4167	0.4242	0.4069	0.3671	0.3877	0.3757	0.3692	0.3510	0.2679	0.2764	0.3075	0.3079
		80	0.4861	0.4697	0.4497	0.4254	0.4321	0.3918	0.3309	0.3269	0.2756	0.2716	0.2743	0.2703
		90	0.5302	0.4880	0.4907	0.4711	0.4574	0.4585	0.3293	0.3253	0.2819	0.2779	0.25943	0.25543
	SDD = 0.07	70	0.4212	0.4275	0.4113	0.3739	0.3920	0.3786	0.3721	0.3537	0.2701	0.2785	0.3091	0.3103
		80	0.4914	0.4734	0.4545	0.4287	0.4368	0.3949	0.3334	0.3294	0.2779	0.2739	0.2755	0.2715
		90	0.5359	0.4918	0.4960	0.4748	0.4624	0.4621	0.3318	0.3278	0.2843	0.2803	0.2605	0.2565

**Table 7:** Values of the third and fifth harmonics for the type 2 surge arrester, three voltage level, humidity, three pollution level and three pollution type in percentage for second scenario.

Current component			I_r3_						I_r5_					
Voltage and SDD		K	360		180		90		360		180		90	
		Humidity %	Exp	Sim-S2	Exp	Sim-S2	Exp	Sim-S2	Exp	Sim-S2	Exp	Sim-S2	Exp	Sim-S2
V_1_ = 16.3	SDD = 0.015	70	0.01940	0.0190	0.0193	0.0177	0.0071	0.0073	0.0318	0.0301	0.0250	0.0256	0.0221	0.0227
		80	0.01583	0.0155	0.0107	0.0107	0.0095	0.0087	0.0356	0.0336	0.0317	0.0321	0.0292	0.0267
		90	0.00121	0.0012	0.0010	0.0010	0.0009	0.0009	0.0378	0.0380	0.0374	0.0356	0.0293	0.0273
	SDD = 0.03	70	0.01955	0.0192	0.0195	0.0177	0.0072	0.0074	0.0321	0.0308	0.0253	0.0260	0.0223	0.0231
		80	0.01602	0.0158	0.0108	0.0108	0.0096	0.0088	0.0360	0.0343	0.0320	0.0326	0.0295	0.0272
		90	0.00123	0.0012	0.0010	0.0010	0.0009	0.0009	0.0382	0.0347	0.0378	0.0361	0.0296	0.0278
	SDD = 0.07	70	0.02269	0.0222	0.0197	0.0177	0.0073	0.0074	0.0325	0.0311	0.0255	0.0262	0.0226	0.0233
		80	0.01619	0.0158	0.0110	0.0110	0.0097	0.0089	0.0364	0.0345	0.0324	0.0327	0.0298	0.0274
		90	0.00124	0.0012	0.0010	0.0010	0.0009	0.0009	0.0386	0.0350	0.0382	0.0363	0.0299	0.0280
V_2_ = 18.3	SDD = 0.015	70	0.01277	0.0121	0.0198	0.0181	0.0073	0.0073	0.0327	0.0305	0.0257	0.0259	0.0227	0.0229
		80	0.01630	0.0156	0.0110	0.0110	0.0098	0.0098	0.0366	0.0340	0.0326	0.0324	0.0300	0.0270
		90	0.00125	0.0012	0.0010	0.0010	0.0009	0.0009	0.0389	0.0384	0.0385	0.0359	0.0301	0.0276
	SDD = 0.03	70	0.01272	0.0123	0.0197	0.0182	0.0073	0.0074	0.0326	0.0312	0.0256	0.0263	0.0226	0.0234
		80	0.01623	0.0159	0.0110	0.0110	0.0097	0.0089	0.0365	0.0347	0.0325	0.0328	0.0299	0.0275
		90	0.00124	0.0012	0.0010	0.0010	0.0009	0.0009	0.0387	0.0352	0.0383	0.0364	0.0300	0.0282
	SDD = 0.07	70	0.01285	0.0123	0.0200	0.0182	0.0074	0.0074	0.0329	0.0315	0.0259	0.0264	0.0229	0.0235
		80	0.01641	0.0160	0.0111	0.0111	0.0098	0.0089	0.0369	0.0349	0.0328	0.0330	0.0302	0.0277
		90	0.00126	0.0012	0.0010	0.0010	0.0009	0.0009	0.0391	0.0354	0.0387	0.0366	0.0303	0.0283
V_3_ = 20.3	SDD = 0.015	70	0.01294	0.0122	0.0201	0.0181	0.0074	0.0074	0.0331	0.0309	0.0260	0.0261	0.0230	0.0232
		80	0.01652	0.0158	0.0112	0.0112	0.0099	0.0099	0.0371	0.0344	0.0330	0.0326	0.0304	0.0313
		90	0.00127	0.0012	0.0010	0.0010	0.0009	0.0009	0.0394	0.0389	0.0390	0.0362	0.0305	0.0279
	SDD = 0.03	70	0.01303	0.0124	0.0202	0.0186	0.0075	0.0074	0.0334	0.0317	0.0262	0.0265	0.0232	0.0236
		80	0.01664	0.0161	0.0113	0.0113	0.0100	0.0100	0.0374	0.0351	0.0333	0.0331	0.0306	0.0279
		90	0.00128	0.0012	0.0010	0.0010	0.0009	0.0009	0.0397	0.0397	0.0393	0.0368	0.0308	0.0285
	SDD = 0.07	70	0.01318	0.0125	0.0205	0.0186	0.0075	0.0075	0.0337	0.0319	0.0265	0.0267	0.0234	0.0238
		80	0.01682	0.0162	0.0114	0.0114	0.0101	0.0101	0.0378	0.0354	0.0336	0.0333	0.0310	0.0281
		90	0.00129	0.0012	0.0011	0.0011	0.0009	0.0009	0.0401	0.0400	0.0397	0.0370	0.0311	0.0287

**Fig. 22:**
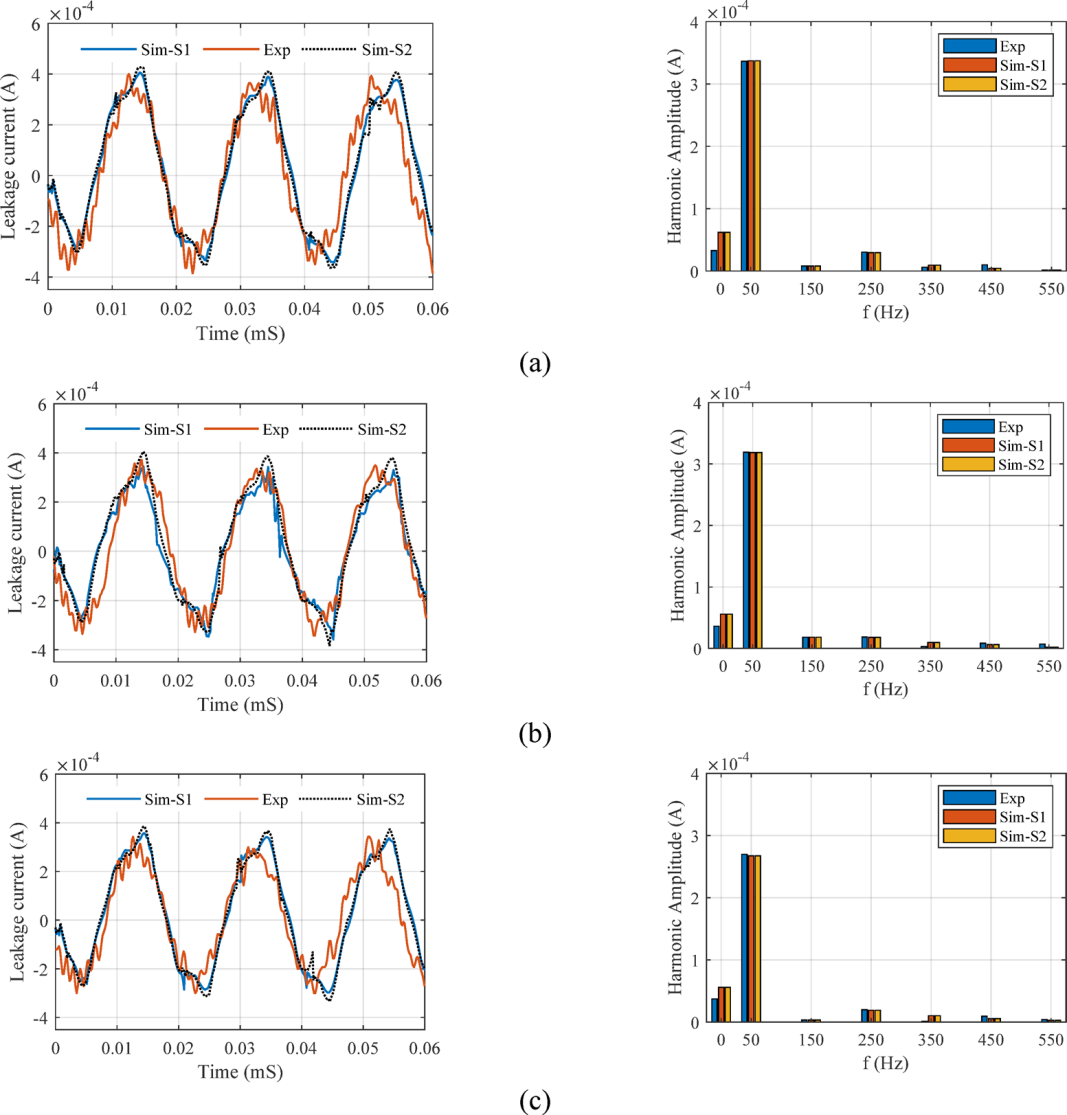
The output obtained from the leakage current of the laboratory test and simulation and the harmonic components of the estimated mode: (**a**) Type = 1, SDD = 0.07, H = 90%, K = 0, V = 16.3 (**b**) Type = 1 SDD = 0.07, H = 90%, K = 0.5, V = 16.3 (**c**) Type = 1, SDD = 0.07, H = 90%, K = 0.75, V = 16.3.

Also, in order to compare the leakage current extracted from FEM simulation and laboratory test in S2, Fig. 22 is provided. For more comparison, the simulated leakage current based on the electrical conductivity characteristics obtained from the first scenario for type 2 surge arrester is also displayed. Examining the results in Table 6 and 7 and Fig. 22 shows the high accuracy of the proposed model in this article. The similarity of the results obtained from the leakage current simulation in the first and second scenario show the high accuracy of the Random Forest method in estimating the pollution layer electrical conductivity characteristic. The matching of the harmonic components obtained from the simulation with the laboratory results is considered as one of the important achievements of this article, which is due to the presentation of an intelligent model in estimating the electrical conductivity of the contaminated layer. The highest error percentage of the estimated value compared to the laboratory test in Table 5 for it, ir1, ir3, ir5 is equal to 9.4, 9.67, 9.36 and 9.85%. These results show the accuracy of the proposed model in estimating the leakage current not only based on the total leakage current but also based on their harmonic components. Also, the average value of error and standard deviation for all the data are equal to 3.72% and 3.39, respectively.

#### Third scenario evaluation

In the third scenario, in order to validate the suggested model, the results are shown for the states that were not present in the proposed model training process. For example, no laboratory test has been done for 85% humidity or 17.3 kV applied voltage. Based on this, the electrical conductivity characteristic in this scenario will be predicted based on the artificial intelligent model with great accuracy.

**Fig. 23:**
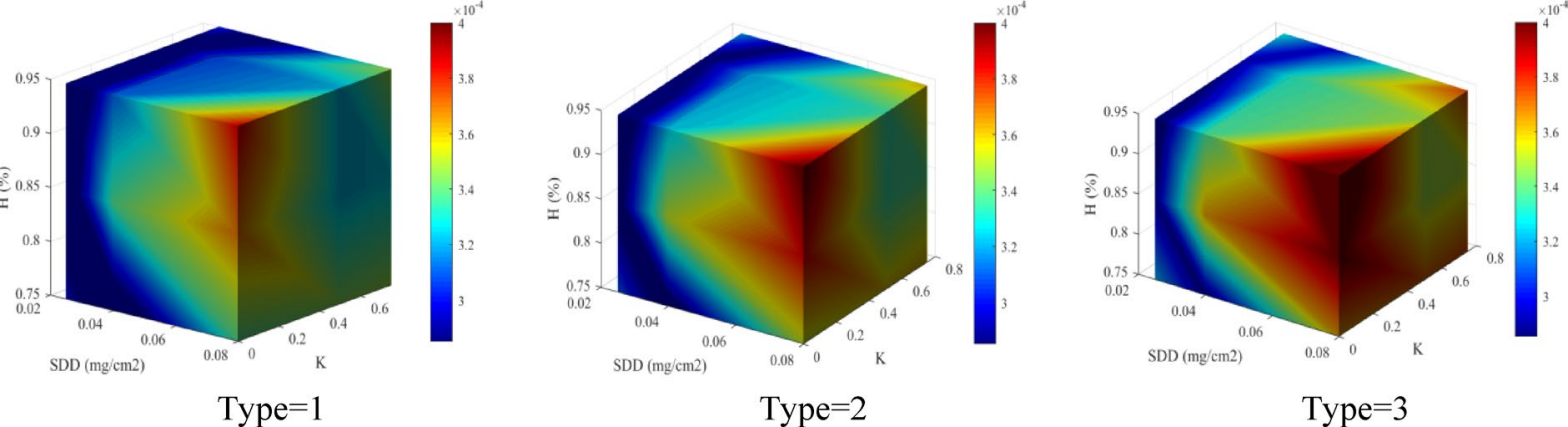
Four-dimensional diagram of the results of first harmonic component according to the third scenario.

To evaluate the performance of the random forest model in S3, the FEM based simulation results is shown in Fig. 23. In the 4D figure, the first harmonic component for three MOSAs is shown with respect to the change of H, K and SDD. Based on the color changes, the MOSA leakage current range can be understood for different parameter variations. The reason for showing this figure is to draw the leakage current in a continuous space so that the effect of different parameters can be better seen, which is the goal of this scenario. For more investigation, the error values of the leakage current harmonics in the conditions corresponding to the third scenario are shown in Table 8. In this table, the error percentage of the harmonic components of the leakage current is shown based on the measured and simulated values at the voltage level of 17.3 kV, SDD 0.025, 0.04 and 0.08 and humidity 75, 85 and 95. The maximum calculated error value for the first, third and fifth components is equal to 9.833, 10.326 and 9.884%, respectively. Also, the average value of the error and standard deviation for all of the data of the leakage current is equal to 3.39% and 2.99, respectively. Using the average value and standard deviation along with the maximum error range and comparing them is very useful and beneficial and shows the high accuracy of the proposed method in simulating the current passing through the surge arrester. Although the maximum error is close to 10%, but according to the average value and standard deviation, it can be found that the simulation error is much less for most of the harmonic components.

**Table 8:** First, second and third harmonic error value for the first surge arrester in percentage.

Current component			I_r1_			I_r3_			I_r5_		
Voltage and SDD		K	360	180	90	360	180	90	360	180	90
		Humidity %	Error (%)			Error (%)			Error (%)		
V_1_=17.3	SDD=0.025	75	6.5164	2.0348	2.5524	3.0168	8.4540	2.6067	5.3153	2.8677	3.0350
		85	3.5119	9.8330	1.5630	1.9056	0	8.5005	5.7286	1.5104	8.6686
		95	1.4064	1.3487	1.6496	0	0	0	0.5841	5.0414	6.9102
	SDD=0.04	75	5.2324	3.4287	0.9039	2.7525	9.3870	2.3942	4.3396	3.1956	3.6611
		85	1.3031	1.3645	1.5526	1.4419	0	8.5032	5.0124	1.7019	8.0304
		95	1.3092	1.3380	1.6422	0	0	0	9.5803	4.7992	6.2327
	SDD=0.08	75	5.2209	3.4012	0.5881	3.3510	10.329	1.6222	4.6157	2.5860	3.2043
		85	1.2931	1.3548	1.5486	1.9456	0	9.1332	5.3912	1.0684	8.4365
		95	1.2995	1.3286	1.6356	0	0	0	9.8836	5.3515	6.6566

## Conclusion

In this article, the estimation of the electrical conductivity of the contaminated layer has been done with the aim of increasing the accuracy of the leakage current modeling of the MOSA under environmental conditions and the effect of uniform and non-uniform pollution. The most important results obtained can be stated as follows.

- In the leakage current modeling, not only the waveform but also the harmonic components have been taken into consideration, which are of great importance in predicting the MOSA performance.
- The random forest method with the least MSE (0.000215) and the most R^2^ (0.999917) is considered as a suitable tool for estimating the electrical conductivity of the contaminated layer.
- Different scenarios have been defined according to the method of modeling the electric conductivity of the contaminated layer. The results have shown that the accuracy of the proposed model has been maintained in the third scenario, which indicates the ability of the artificial intelligence method to predict conditions that the random forest method has not been trained on.
- By using FAM, in addition to the possibility of modeling the environmental conditions and electrical parameters of the MOSA with high accuracy, the development of the proposed model based on the changes in the structure of the MOSA and the operating conditions is easy.
- Average values and standard deviation of the error obtained from the proposed model and laboratory results for harmonic components of leakage current in the first scenario is 3.11% and 3.22, in the second scenario 3.72% and 3.39 and in the third scenario is 3.39% and 2.99, respectively. This issue shows the ability of the FEM model in accurately reproducing the characteristics of the leakage current and its harmonic components in different conditions.
- The results of the evaluation of the artificial intelligence method have shown that the selection of the input parameters and the appropriate implementation method can solve the problems caused by the number of laboratory data, which has been raised as a problem in this field, to a great extent.

## Data Availability

All of the data are included within the manuscript. Also, more information available from the corresponding author on reasonable request.
